# c-Src kinase inhibits osteogenic differentiation via enhancing STAT1 stability

**DOI:** 10.1371/journal.pone.0241646

**Published:** 2020-11-12

**Authors:** Zahra Alvandi, Michal Opas

**Affiliations:** 1 Department of Laboratory Medicine and Pathobiology, University of Toronto, Toronto, ON, Canada; 2 Department of Vascular Biology, Boston Children’s Hospital, Boston, MA, United States of America; 3 Department of Surgery, Harvard Medical School, Boston, MA, United States of America; Universite de Nantes, FRANCE

## Abstract

The proto-oncogene Src is ubiquitously expressed and is involved in cellular differentiation. However, the role of Src in embryonic stem (ES) cell osteogenic differentiation is largely unknown. Using the small molecule inhibitor PP2, c-Src specific siRNAs, and tet-inducible lentiviral vectors overexpressing active c-Src, we delineated an inhibitory role of c-Src in osteogenic differentiation of mouse embryonic stem cells (mESCs) and mouse MC3T3-E1s preosteoblasts. Active c-Src was shown to restrict the nuclear residency of Runt-related transcription factor 2 (Runx2) and its transcriptional activity with no detectable effect on Runx2 expression level. Furthermore, we showed Signal Transducer and Activator of Transcription 1 (STAT1) was indispensable to the inhibitory role of c-Src on Runx2 nuclear localization. Specifically, higher levels of active c-Src increased STAT1 half-life by inhibiting its proteasomal degradation, thereby increasing the cytoplasmic abundance of STAT1. More abundant cytoplasmic STAT1 bound and anchored Runx2, which restricted its nucleocytoplasmic shuttling and ultimately reduced Runx2 transcriptional activity. Collectively, this study has defined a new mechanism by which c-Src inhibits the transcriptional regulation of osteogenesis from mESCs *in vitro*.

## Introduction

Src family kinases play crucial roles in regulating cellular adhesion, growth, migration and differentiation [1]. c-Src has been shown to increase the rate of bone resorption in mice [2, 3] and to decrease osteoblast differentiation *in vitro* [4, 5]. More recently, it was shown that inhibition of c-Src activity in adult mice increases bone mass at least in part by stimulating osteoblast differentiation [6]. However, while the inhibitory role of c-Src in osteogenesis has been studied, the role of c-Src in osteoblast differentiation of embryonic stem cells (ESCs) remains unclear.

Osteoblast differentiation progresses through a sequence of steps comprising formation of immature and mature osteoprogenitors, preosteoblasts, mature osteoblasts and osteocytes [7, 8]. Differentiation of osteoblasts from mesenchymal osteo-progenitors involves proliferation, maturation, extracellular matrix (ECM) development and mineralization, which are linked to variable gene expression of osteogenic markers [9, 10]. Osteocalcin (OC) is carboxylated and released from osteoblasts and deposits in the bone matrix [11]. Enrichment of the ECM scaffold with OC promotes deposition of minerals, mainly calcium phosphate [10]. ECM mineralization can be assessed by von Kossa or Alizarin Red S (ARS) staining for evaluation of osteogenic differentiation progression.

Osteo-chondroprogenitor cells become committed to the osteoblastic cell lineage during mesenchymal condensation under the effect of the runt-domain related transcription factor 2 (Runx2) [12, 13]. Runx2 is a master regulatory gene required to control the expression of several key osteogenic transcription factors or proteins such as collagen I (COL I), bone sialoprotein II (BSP II), and OC during ES cell osteogenic differentiation [10, 11, 14, 15]. It is currently not completely understood how Runx2 is regulated during the development of the skeletal system. Another transcription factor participating both in osteoclastogenesis and osteoblast differentiation is STAT1 [16, 17], the subcellular distribution of which, akin to many other transcription factors including Runx2 [18–21], affects differentiation outcome [22–24].

In the present study, we showed that c-Src activity inhibits osteogenesis from ESCs via Runx2 and that STAT1 is necessary for c-Src inhibitory effect on Runx2 transcriptional activity by acting as a cytoplasmic anchor of Runx2. Finally, we provided evidence that inhibition of c-Src increases STAT1 proteasomal degradation thus leaving Runx2 free to undergo trafficking to the nucleus. These findings collectively suggest that decreased interaction of Runx2 with STAT1 in the cytoplasm in response to c-Src inhibition allows for increased nuclear residency of Runx2, which dramatically enhances its transcriptional activity. Therefore, we propose a novel mechanism in which c-Src regulates mESCs osteogenic differentiation via the effects on Runx2 subcellular trafficking.

## Materials and methods

### Cell culture

Mouse ES cells R1 derived from J1 129/mice [25] were maintained in their undifferentiated status in Dulbecco’s modification eagle’s medium 1X (DMEM, Catalog No. 319-005-ES) supplemented with 10% FBS (FBS, Premium, Catalog No. 088150), 1% MEM non-essential amino acids (MEM NEAA 100X, Gibco, Catalog No. 11140050), 10 nM 2mercaptoethanol (Bioshop, Catalog No. MER 002), and LIF. Drops containing 250 cells per 25 μl DMEM supplemented with 20% FBS were placed on the lids of tissue culture dishes for 3 days to form EBs. After 3 days EBs were transferred into the floating cell culture dishes containing medium supplemented with 0.1 μM retinoic acid (Sigma, Catalog No. R2625) for 2 days. On day 5, the EBs were plated in tissue culture dishes coated with 0.1% gelatin. On day 6, the differentiation medium was supplemented with 50 μg/ml L-ascorbic acid (Sigma, catalog No. A5960) and 10 mM β-glycerophosphate disodium salt hydrate (Sigma, catalog No. G9422) to promote osteogenic differentiation. 100 nM dexamethasone (Sigma, Catalog No. D4902) was added on day 10 to further enrich cells of the osteogenic lineage. The medium was changed every 2 days for the entire 21 days of differentiation.

MC3T3-E1 subclone 4 (ATCC^®^ CRL2593™) were purchased from ATCC. Cells were maintained in a-MEM with ribonucleotides, deoxyribonucleosides, 2 mM L-glutamine and 1 mM sodium pyruvate, but without ascorbic acid (Gibco, Custom Product, Catalog No. A1049001) supplemented with 10% (v/v) FBS. For osteogenic differentiation, the cells were cultured in a-MEM supplemented with 10% FBS, 50 mg/ml ascorbic acid, and 10 mM βglycerophosphate disodium salt hydrate for 21 days. Medium was renewed every 3 days.

### Inhibition assay

c-Src inhibitor PP2 (Calbiochem, Catalog No. 529573) and negative control PP3 (Calbiochem, Catalog No. 529574) were applied at concentrations described by us previously [26, 27] and in ES cells specifically [28] based on survey by Bain *et al*. [29] wherever indicated. For multiple days treatment, media was replaced with fresh inhibitor containing media every day during the treatment period. For protein half-life assay, Cycloheximide (Sigma-Aldrich, Catalog No. C7698) was applied at 10 μM concentration for the indicated times. Proteasome inhibitor MG132 was purchased from Millipore (Catalog No. 474790).

### Real-time PCR analysis

The Qiagen RNeasy Mini Kit (Qiagen, Catalog No. 74134) was used to extract total RNA according to the manufacturer’s instructions. 500 ng RNA was reverse transcribed to cDNA using iScript cDNA Synthesis Kit (Bio-Rad, Catalog No. 1708890) in a total reaction volume of 20 μl. Real-time qPCR analysis was performed in Bio-Rad’s CFX384 Touch™ detection system. The cDNA levels were normalized against L32 gene. The primers sequences used in this study are listed in S1 Table.

### Immunoprecipitation assay

Cell extracts were harvested in a Pierce IP Lysis Buffer® (25 mM Tris-HCl pH 7.4, 150 mM NaCl, 1% NP-40, 1 mM EDTA, 5% glycerol). 500 μg cell extracts were incubated with 1 μg anti-RUNX2 (M-70, Santa Cruz, Catalog No.sc-10758), or STAT1 (Cell signaling, Catalog No.9172S) at 4° C overnight. Immune complexes were recovered with True Blot® anti-rabbit Ig beads (Rockland, Catalog No.00-8800-25) and subjected to SDS-PAGE.

### Chromatin Immunoprecipitation (ChIP)

The protein-DNA complexes were cross-linked with 1% formaldehyde (10 min, RT). Cells were then resuspended in ChIP lysis buffer (50 mM Tris-HCl pH 8, 150 mM NaCl, 1 mM EDTA, 1% Triton X-100, 0.1% Na-deoxycholate containing protease inhibitor cocktail (Sigma-Aldrich, Catalog No. P8340) and 1 mM DTT. Cells were sonicated 10 times for 15 seconds each time and soluble chromatin was pre-cleared with agarose beads for 1h. An aliquot of the pre-cleared chromatin served as an input. The supernatant was incubated overnight with 1 μg of Runx2 (M-70, Santa Cruz, Catalog No.sc10758), ChIP grade Rabbit IgG, polyclonal isotype control (Abcam, Catalog No. ab171870), and RNA polymerase II (Abcam, Catalog No. ab5131) at 4° C and incubated with the agarose beads for 2 hours the following day. Beads were then washed for 10 minutes once in 1mL of low-salt immune complex wash buffer (20 mM Tris-HCl [pH 8.0], 150 mM NaCl, 2 mM EDTA, 1% Triton X-100, 0.1% SDS), once in 1 ml of high-salt immune complex wash buffer (20 mM Tris-HCl [pH 8.0], 500 mM NaCl, 2 mM EDTA, 1% Triton X100, 0.1% SDS), once in 1 ml of LiCl buffer (20 mM Tris-HCl [pH 8.0], 250 mM LiCl, 1 mM EDTA, 1% NP-40, 1% Na-deoxycholate), and twice with 1 ml of TE (10 mM Tris-HCl [pH 8.0], 1 mM EDTA) in the order that are explained. Protein-DNA complexes were eluted in 100 ul of fresh ChIP elution buffer (1% SDS, 0.1M NaCO3) for 1hr at 37°C in two settings of 50 μl of elution buffer for 30 minutes. Cross-linked eluted complexes were then reversed by overnight incubation using 0.2 M NaCl at 65°C. The recovered DNA was then treated with 1 μg of RNase A for 30 minutes at 37°C and 10 μg of Proteinase K for 2 hours at 45°C. DNA was purified using a PCR purification kit (QIAGEN, Catalog No. 28104) and eluted in 100 ul of DNase-RNase free water. ChIP DNA was amplified by qPCR using COL1A1, OC, BSPII, and COL2A primer pairs. A list of ChIP primers is provided in S2 Table.

### Immunoblot analysis

20 μg cell lysates collected in CST lysis buffer (Catalog No. 9803) were separated by SDS-PAGE and transferred to nitrocellulose membrane. For immunoblotting, anti-STAT1 (Cell Signaling, catalog No.9172S), anti-GAPDH (Cell Signaling, Catalog No.2118S), anti-c-Src (Cell Signaling, Catalog No. 2108), anti-p-Y416-c-Src (Cell Signaling, Catalog No.2101S), anti- Runx2 (Cell Signaling, Catalog No.8486S) antibodies were applied. The secondary antibody was goat polyclonal secondary antibody to rabbit IgG–H&L (HRP) (Abcam, Catalog No. ab6721) or rabbit polyclonal secondary antibody to mouse (HRP) (Abcam, Catalog No. ab6728). The secondary antibody for immunoblot analysis of IP products was Rabbit True Blot® anti Rabbit IgG (HRP) (Rockland, Catalog No.18-8816-31).

### Mineral deposition assay

4 mM Alizarin Red S (Sigma, Catalog No. A5533) was prepared in distilled water and the pH was adjusted to 4.0 using 10% ammonium hydroxide. Cultures were fixed with 4% formalin for 15 minutes, triple washed with dH2O, and stained with Alizarin Red S for 20 minutes. After removal of unincorporated excess dye with distilled water, the absorbed stains by mineral nodules were then dissolved in 10% acetic acid and absorbance of triplicates were read at 405 nm. The concentration of ARS was calculated using the equation of the trend line of ARS standard concentrations.

### Immunofluorescence (IF) staining

Nodules on coverslips were rinsed 3 times with PBS, fixed in 3.7% formaldehyde for 15 minutes, washed three times for 5 minutes each time in PBS, and permeabilized with 0.1% Triton X-100 in buffered containing 100 mM 1,4 piperazinediethanesulfonic acid, 1 mM EGTA, and 4% (wt/vol) polyethylene glycol 8000 (pH 6.9) for 2 minutes. After 3X washing with 1X PBS for 5 minutes, they were blocked by 1% BSA, 22.52 mg/mL glycine in PBST (0.1% Tween 20) for 30 minutes. After removal of blocking buffer nodules were incubated with 1:1000 anti-Runx2 (Abcam, Catalog No. ab76956) at 4°C overnight. Next day nodules were washed in PBS (3 times for 5 min), incubated with the secondary antibody fluorescein (FITC)-conjugated donkey anti-mouse (Jackson ImmunoResearch, Catalog No. 715-096-151) for 1hour at room temperature, washed 3 times in PBS for 10 minutes and mounted in Pro® Gold Antifade Mountant (Thermo Fisher Scientific, Catalog No. P36931). Imaging was performed using confocal LSM800 microscope.

### Subcellular fractionation

Nuclear and cytoplasmic fractions were prepared using the Nuclear Extraction Kit (Millipore, Catalog No. 2900) as per manufacturer’s instruction.

### Transfection

c-Src siRNAs (Catalog No. S238007, S238008, and S238009), STAT1 siRNA (Catalog No. AM16708), GAPDH siRNA (Catalog No. 4390850), and Ambion® Silencer® Negative Control #1 (Catalog No. 4390843) were purchased from Thermo Fisher Scientific. MC3T3 E1 cells were transfected with diluted siRNA and lipofectamine RNAiMax reagent (Thermo Fisher Scientific, Catalog No. 13778030) according to the manufacturer instructions. For ESCs transfection, Neon™ transfection system was used following the recommended protocol for mESC transfection.

### Lentiviral transduction

Lenti-X 293T cell line (Clontech, Catalog No. 632180) was used for lentivirus production. The cells were plated at 70% confluency the day before transfection on 100 mm culture dishes. 7 μg lentiviral vectors containing active mouse c-Src (LV[Exp]-Tet3G:T2A:Puro TRE3G>mSrc [NM_009271.3]*del:3xGGGGS:EGFP, VectorBuilder), or m-Cherry vector were diluted in 600 μl of nuclease free sterile water. Dilute DNA was then added to one tube of LentiX™ Packaging Single Shots Ecotropic, (TaKaRa, Catalog No. 631278), vortexed and the mix was then incubated at room temperature for 10 minutes prior to addition to Lenti-X 293T cells in 5 mls of serum-free, antibiotic-free DMEM (Sigma, Catalog No. D5671). 24 hours post-transfection 5 ml fresh serum-free, antibiotic-free media was added to the culture. 48 hours after transfection supernatant containing viral particles was harvested and filtered through 0.45 μm filters Millipore (Catalog No. SLHA033SS) and aliquots were stored at -80°C.

For transduction, media containing 10% Tet System Approved FBS (Takara, Catalog No. 631106) was prepared. 10 μl of Ecotropic Receptor Booster (TaKaRa, Catalog No. 631471) along with the media containing 4 μg/ml polybrene (PB = hexadimethrine bromide; Sigma, Catalog No. H-9268) was added to 60% confluent 6 well-plate cultures of MC3T3-E1s. Plates were then centrifuged at 1200g for 20 minutes and incubated at 37°C for 2 hours. Media was replaced with 2 ml complete PB containing media for all wells and viral containing supernatant at MOI = 10 was added to each well. After 16 hours, the virus containing PB media was replaced with 2 ml complete media. Next day, cells were treated with 2.5 μg/ml puromycin (Sigma, Catalog No. P-8833) to select transduced cells for 5 to 7 days. For doxycycline-induced protein over-expression, the transduced cells were incubated in 10% FBS DMEM containing 2 μg/mL doxycycline (Sigma, Catalog No. D-9891) for the indicated days. The medium was refreshed every 24 hours.

### Statistical analysis

All data were analyzed using Prism software. Statistical differences between two groups were analyzed by two-tailed Student’s t test. Data are represented as mean ±SD. A value of *p*<0.05 was considered significant. Multiple group statistical analysis was performed using one-way and two-way ANOVA followed by Bonferroni or Tukey where indicated.

## Results and discussion

### c-Src inhibition in early days of mESCs osteogenic differentiation enhances osteogenesis

**Fig 1:**
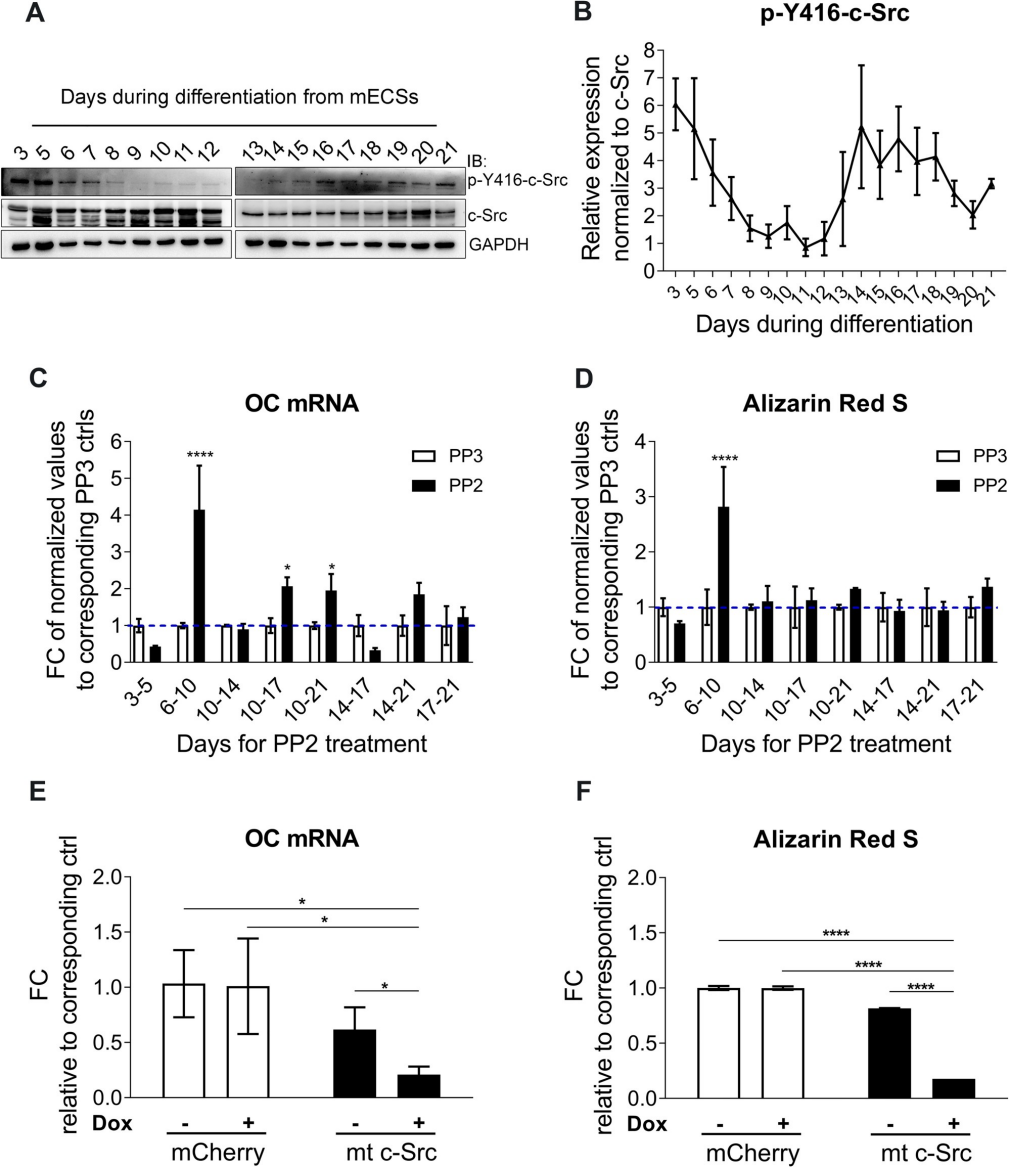
c-Src inhibition enhances osteogenic differentiation in mESCs. (A) c-Src activity during 21 days of osteogenic differentiation in mESCs. p-Y416-c-Src level and total level of c-Src are analyzed by immunoblotting. GAPDH served as loading control. (B) p-Y416-c-Src band densities are quantified using ImageJ. Normalized values to corresponding total c-Src and GAPDH controls are graphed. (C) Total RNA of day 21 osteo-nodules is subjected to qPCR analysis using OC primer pairs. Fold change in OC expression is calculated by normalizing PP2 treated values to their corresponding PP3 control. Values representing the mean (±SD) of triplicates are graphed and subjected to one-way ANOVA; F (15,32) = 15.85, *p*<0.0001. Bonferroni's multiple comparisons test indicated significant differences between PP2 and PP3 treated cells for day 6–10 (*p*<0.0001), day 10–17 (*p* = 0.0161), and day 10–21 (*p* = 0.0422). (D) Day 21 osteo-nodules were stained by ARS method. Absorbed stains were extracted and their concentration were quantified and normalized to their corresponding PP3 controls. Data shown represent the mean (±SD) of triplicates and subjected to one-way ANOVA; F (15,32) = 8.651, *p*<0.0001. Bonferroni's multiple comparisons test revealed significant differences between PP2 and PP3 treated cells for day 6–10 with *p*<0.0001. (E) Day 14 qPCR analysis for OC mRNA expression in MC3T3-E1s upon overexpression of mt c-Src. Values from doxycycline- free condition were used to normalize the induced condition. Data representing mean of triplicate values (±SD) are graphed. (F) Day 14 differentiated MC3T3-E1 cells for the indicated conditions were subjected to ARS for mineralization assessment and concentrations of extracted Alizarin Red S were quantified. Mean of triplicates (±SD) are graphed and subjected to one-way ANOVA; F (3,8) = 3066, *p*<0.0001. Bonferroni post hoc analysis was ****p<0.0001 for the indicated comparisons.

Osteogenic differentiation from mESCs was carried out *in vitro* based on our optimized 21-day protocol and assessed using qPCR analysis of osteogenic transcription factors including Runx2 and osterix (Osx) and markers including BSPII, COL1A1, and OC (S1 Fig). To study the role of c-Src in mESCs osteogenic differentiation, we first evaluated the activity of c-Src, as measured by phosphorylation levels on tyrosine 416 (p-Y416) throughout the 21 days of osteogenic differentiation of R1 cells. The activity of c-Src dropped progressively during the first 5 days of differentiation reaching minimum at approximately mid differentiation (Fig 1A and 1B). Dynamic changes in SFKs expression and activity during EB formation in human ES cells was noted in former studies suggesting their potential role in differentiation [30]. Down-regulation of c-Src kinase activity after induction of osteogenic differentiation is consistent with earlier studies supporting the inhibitory role of c-Src in osteogenic differentiation. Later increase in c-Src activity, starting from day 14, however, may suggest that osteogenic differentiation may benefit from c-Src activity at later stages. One study showed that c-Src interacts with and phosphorylates osterix and subsequently increases its stability and transcriptional activity [31]. To better understand the significance of c-Src during osteogenic differentiation, we inhibited c-Src with PP2 (10 μM) for different periods along the differentiation timeline. Choice of inhibitor and applied concentration was based on the assessments of multiple Src inhibitors including PP1, PP2, SrcI-1 (S2A and S2B Fig). DMSO as solvent negative control and PP3 (inactive analog of PP2;10 uM) were deployed in the subsequent assays. The effect of DMSO, PP3, and PP2 on c-Src phosphorylation at Y416 was tested at multiple concentrations in an independent assay which confirmed PP2 at 10 uM dose efficiently inhibits c-Src activity and that DMSO and PP3 were ineffective (S2C Fig). A scheme summarizes the time periods included in this study in which Src was inhibited throughout the 21 osteogenic differentiation of mESCs (S2D Fig). Analysis showed that the most efficacious Src inhibition was between day 6 and day 10 evident by a significant increase in both OC mRNA abundance—which is strongly expressed in more mature osteoblasts and is used as a specific marker of osteogenic differentiation (Fig 1C), and mineralization (Fig 1D and S2E Fig). The indicated timing of PP2 administration was consistent with natural downregulation of p-Y416-c-Src, commencing in the early days of mESCs osteogenic differentiation. OC mRNA expression was also increased when c-Src was inhibited for relatively longer periods between day 10 to 17, and 10 to 21, although not as profoundly. However, this was not reflected in the corresponding mineralization level. Inhibition of c-Src between day 3 to 5 reduced day 21 OC expression and mineralization of the osteo-nodules. It is possible that inhibition of c-Src during this period interferes with cell division of proliferating EBs. This subsequently reduces the osteogenic differentiation due to the decreased number of cells that were initially available to go through the differentiation process. This is consistent with studies that suggest c-Src activity promotes proliferation and initiation of differentiation [32]. Another possibility to consider for the time periods which showed some level of reduction in OC mRNA when treated with PP2 would be that ES osteogenic differentiation in culture is heterogeneous. Therefore, the overall differentiation outcome of a population of cells at proliferation phase were compromised. Moreover, the potential role of c-Src in regulation of osterix transcriptional activity [31] combined by osterix expression pattern in our setting implies that c-Src inhibition of osterix could play a role in down regulation of osteogenic differentiation during some of the indicated time periods.

To investigate the specificity of c-Src’s effect, c-Src specific siRNAs were applied. However, extraordinarily low transfection efficiency in R1 ES cells (S3A–S3C Fig) led us to use MC3T3-E1 pre-osteoblasts instead. Applied c-Src specific siRNAs in MC3T3-E1 cells efficiently down regulated c-Src expression and activity (S4A Fig), increased both OC mRNA abundance (S4B Fig) and mineralization (S4C Fig) at day 14 and day 21 of osteogenic differentiation. SFK inhibitor has been shown to enhance osteoblast differentiation in MC3T3-E1 mainly through inhibition of c-Src activity [33]. However, in our study the observed effect was not as profound when compared to ESCs. The mechanisms that contribute to such differences remain to be determined. One explanation however, could be that MC3T3-E1 cells are inherently further advanced to begin with in terms of osteogenic differentiation [34] than ESCs and hence inhibition of c-Src would not be as efficacious as in MC3T3-E1 cells.

The effect of PP2 on expression levels of OC (S4D Fig) and mineralization level (S4E Fig) in MC3T3-E1 cells was concentration-dependent with 10 μM being most efficacious. Interestingly, the levels of non-specific osteogenic marker COL2A remained unchanged indicating specificity (S4F Fig). To examine the effect of active c-Src overexpression, MC3T3-E1 cells were transduced with MOI = 10 of viral containing supernatants obtained from transfected Lenti-X 293T cells. Transfection efficacy was examined by microscopy imaging after 48 hours (S5A Fig). M-Cherry viral particles were used to transduce MC3T3-E1s as negative control. MC3T3-E1s were transduced by dox-inducible lentiviral particles overexpressing mutant c-Src (mt c-Src). Transduction efficiency was examined and is shown in S5B Fig. qPCR analysis of day 14 lysates revealed a significant decrease in OC mRNA expression when compared to empty vector controls (Fig 1E). ARS also confirmed lower level of mineralization when mutant c-Src was overexpressed (Fig 1F). Collectively, these results provide evidence that inhibition of c-Src activity in early ES osteogenesis leads to accelerated osteogenic differentiation, increased matrix production and mineralization *in vitro*.

### c-Src kinase activity inhibits Runx2 nuclear localization

**Fig 2:**
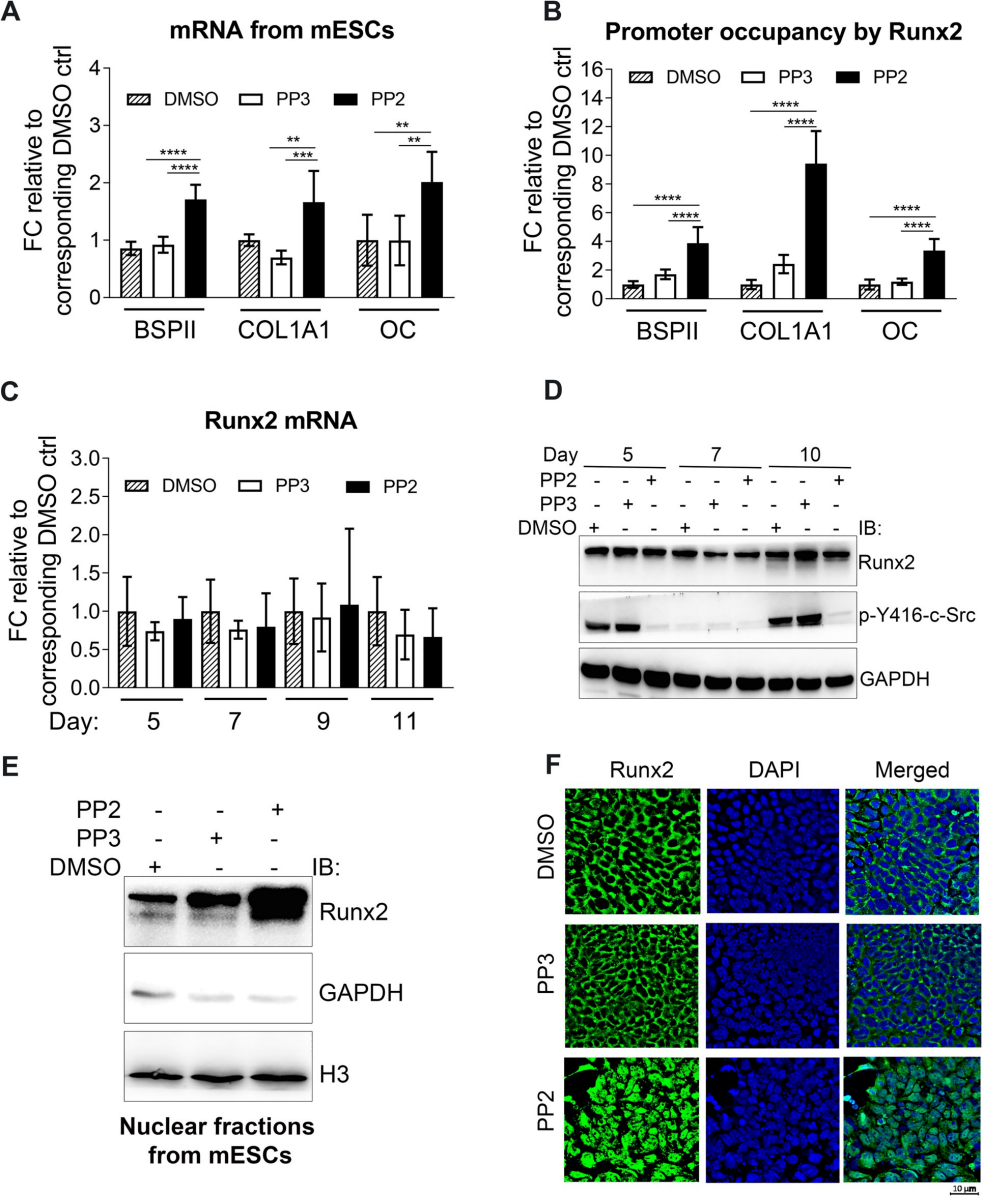
Runx2 nuclear localization and transcriptional activity are increased when c-Src is inhibited in mESCs. (A) Real-time analysis of Runx2 target genes expression including BSPII, COL1A1, and OC in response to c-Src pharmacological inhibition. Day 10 differentiating EBs were treated with either PP2 (10 μM) or PP3 (10 μM) and DMSO as controls for 24 hrs. Data are expressed as means (±SD) of triplicates. One-way ANOVA is conducted; BSPII: F (2,15) = 42.41, *p*<0.0001, COL1A1: F (2,15) = 13.52, *p* = 0.0004, and OC: F (2,14) = 8.428, *p* = 0.004. Bonferroni's multiple comparisons test results for PP2 vs. DMSO and PP2 vs. PP3 treated results are as follows; BSPII: *p*<0.0001 for both, COL1A1: *p* = 0.0098 and *p* = 0.0004, and OC: *p* = 0.0087 and *p* = 0.0084. (B) Promoter occupancy of Runx2 target genes were assessed by ChIP analysis in mESCs. Day 10 differentiating EBs were treated with either PP2 (10 μM) or PP3 (10 μM) and DMSO as controls for 24 hrs. Values were normalized to their corresponding 1% inputs and then normalized to their corresponding DMSO controls. Data shown represent the means (±SD) of triplicates. One-way ANOVA is conducted; BSPII: F (2,24) = 42.95, *p*<0.0001, COL1A1: F (2,24) = 28.18, *p*<0.0001, and OC: F (2,24) = 56.17, *p*<0.0001. Bonferroni's multiple comparisons test results for PP2 vs. DMSO and PP2 vs. PP3 treated results are as follows; BSPII: *p*<0.0001 for both, COL1A1: *p*<0.0001 for both, and OC: *p*<0.0001 for both. (C) Real-time qPCR analysis of Runx2 expression upon c-Src inhibition in mESCs during osteogenic differentiation at day 5, 7, 9, and 11. Data shown represent the means (±SD) of triplicates of three independent experiments. mRNA of each day was normalized to the corresponding DMSO. (D) Protein level of Runx2 with or without c-Src inhibition. Differentiating EBs were treated with DMSO, PP3, or PP2 (10 μM) at different days including day 5, 7, and 9 of osteogenic differentiation and Runx2 protein levels were analyzed by immunoblot. GAPDH served as loading control. (E) Runx2 nuclear fractions in mESCs at day 10 of osteogenic differentiation when c-Src is inhibited. Day 10 differentiating EBs were treated with either PP2 (10 μM) or PP3 (10 μM) and DMSO as controls for 2 hrs. Cell lysates were subjected to immunoblot analysis with GAPDH (cytosolic marker) and H3 (nuclear marker) as loading controls. (F) Runx2 subcellular localization at day 10 of osteogenic differentiation. Day 10 differentiating EBs were treated with either PP2 (10 μM) or PP3 (10 μM) and DMSO as controls for 2 hrs. Cells were then fixed and subjected to immunofluorescence staining using Runx2 antibody (Green). DAPI was used to stain nuclei (blue). *p< 0.05, **p<0.01, ***p<0.005, and ****p<0.0001.

The early commitment of stem cells to osteoblast lineage requires Runx2 [11]. Considering the significance of c-Src in the early days of osteogenesis, we investigated the potential role of c-Src in the regulation of Runx2 transcriptional activity. Hence, differentiating mESCs were treated for 24 hours with PP2 or a negative control, PP3 (the inactive analog of PP2) or DMSO. Lysates were subjected to qPCR using specific primers listed in S1 Table. Inhibition of c-Src activity by PP2 increased mRNA expression of BSPII and COL1A1 by almost 1.5-fold (*p*<0.01, and *p*<0.001, respectively) and of OC by more than 2-fold (*p*<0.01) when compared to DMSO (Fig 2A). Next, ChIP analysis was performed to test the transcriptional activity of Runx2 at the Runx2 target genes promoters. This revealed that when c-Src activity is inhibited, the promoter occupancy of the COL1A, BSPII, and OC by Runx2 are significantly increased by 3.9, 9.3, and 3.4-fold, respectively (Fig 2B). Furthermore, Runx2 expression in response to Src inhibition was examined in differentiating EBs at day 5, 7, 9, and 11 using qPCR and WB analysis. No significant difference was observed (Fig 2C and 2D). c-Src-depleted MC3T3-E1 cells also showed no detectable modification in Runx2 protein levels (S4A Fig).

What could account for the observed promotion of osteogenic differentiation by c-Src inhibition in face of a lack of discernible effects on Runx2 expression during differentiation? To address this, we postulated that the observed increase in OC expression might result from increased Runx2 nuclear localization. Western blot analysis of Runx2 nuclear fractions revealed that c-Src inhibition at day 10 of osteogenic differentiation from mESCs significantly enhanced Runx2 nuclear localization (Fig 2E). This may indicate that inhibition of c-Src activity most likely does not affect the number of osteoprogenitors at least not for the duration of time that we studied, but most likely increases osteogenic differentiation by enhancing Runx2 transcriptional activity. Similar results were obtained when c-Src depleted MC3T3-E1s were analysed (S6A Fig). Finally, immunofluorescence localization clearly demonstrated that the nuclear residency of Runx2 significantly increased when c-Src was inhibited by PP2 (Fig 2F). Regulation of Runx2 transcriptional activity by c-Src has been reported previously where showed the endogenous YAP interacts with the native Runx2 protein and suppresses Runx2 transcriptional activity in a dose-dependent manner in Rat osteosarcoma ROS 17/2.8 [35]. However, the induction of OC when ROS 17/2.8 cells were treated with 5 uM of PP2 was almost 3 folds higher comparing to the cells treated with PP2 (1 uM), Src DN, or Yap DN (Fig 6C in Zaidi *et al*.). Collectively, this could indicate that there are other mechanisms under which SFKs inhibit osteogenic differentiation that could be suppressed using higher concentrations of PP2. In a recent study, we confirmed that higher c-Src activity in mESCs reduces Runx2 nuclear localization and that PP2 and SrcI-1 increase intranuclear presence of Runx2 [36]. Cyril Thouverey *et al*. showed that SU6656, a selective inhibitor of SFK increases osteoblast differentiation from MC3T3-E1 by inhibiting c-Src and Yes [6]. Zhang *et al*. demonstrated that the differentiation-promoting activity of c-Src is antagonized by c-Yes in mES cells, despite their very close phylogenetic relationship [30]. Therefore, it might be argued that c-Src and Yes may not be partnering in mouse ES differentiation and that may be the regulation of Runx2 localization is not solely depend on YAP pathway in mESCs. However, differences in the cellular models used in each study (mESCs in the current study and Rat osteosarcoma ROS 17/2.8 in the former study) limit further speculations.

### c-Src regulates Runx2 subcellular localization through STAT1

**Fig 3:**
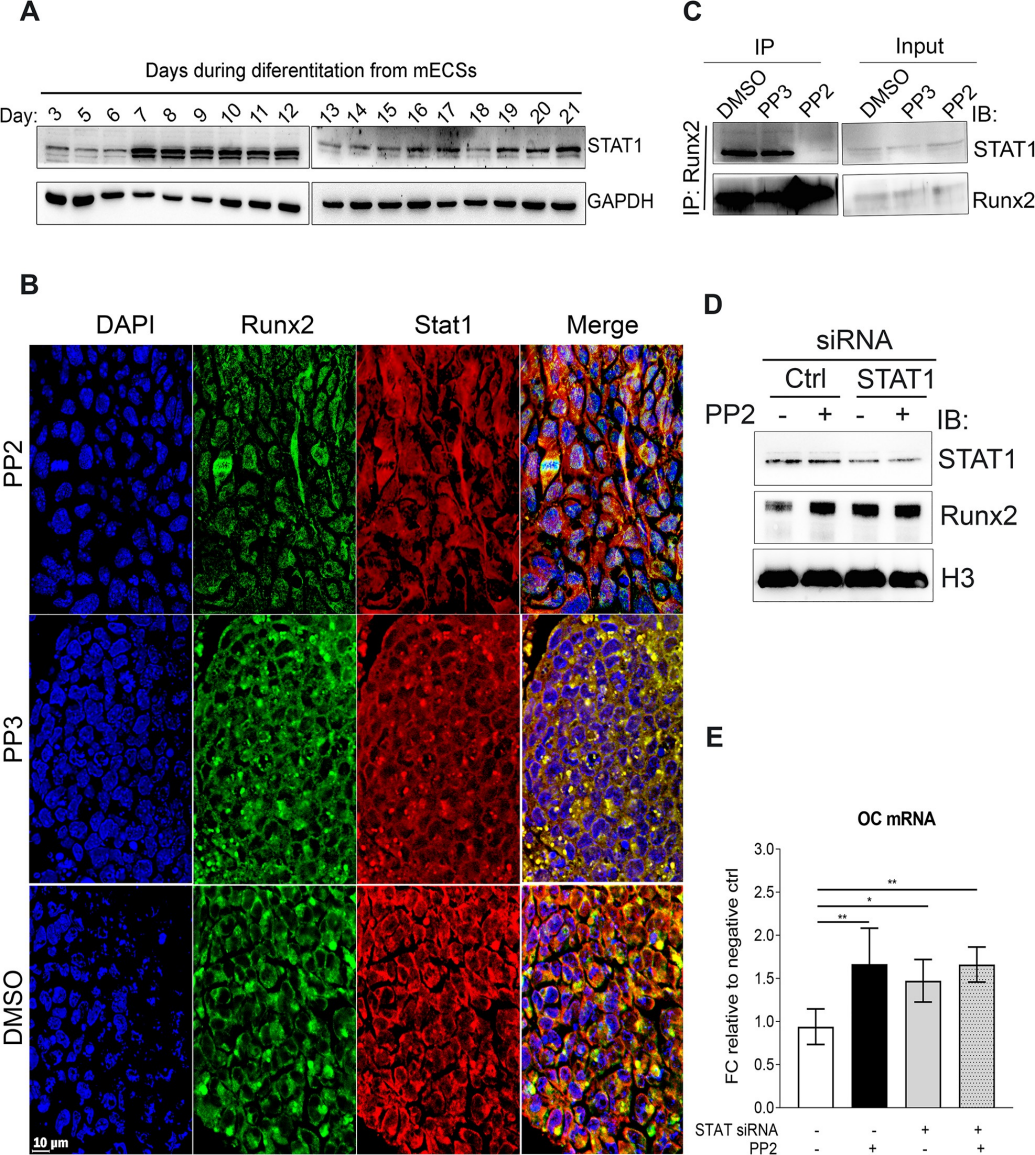
c-Src regulates Runx2 subcellular localization through STAT1. (A) STAT1 expression throughout osteogenic differentiation from mESCs. Differentiating EBs from day 3 to 21 have been subjected to WB analysis using STAT1 antibody. GAPDH served as loading control. (B) Subcellular localization of Runx2 and STAT1 in the presence and absence of c-Src activity. D15 differentiating EBs underwent treatment with DMSO, PP3, and PP2 for two hours and were fixed and subjected to IF staining using Runx2 (green) and STAT1 (red) antibodies. DAPI was used to stain nuclei (blue). (C) Runx2 interaction with STAT1 in the presence or absence of c-Src activity. Differentiating EBs were treated with PP2 (10 μM) or PP3 (10 μM) or DMSO as controls for 2 hrs. Cell lysates were then subjected to IP assays and analyzed by immunoblot. (D) c-Src effect on Runx2 in STAT1 depleted MC3T3-E1. Cells were transfected with STAT1 specific siRNA along with Silencer™ select negative control siRNA. 48 hours post transfection cells were treated either with PP2 (10 μM) or DMSO for 2 hours prior to fractionation assay. Nuclear fractions were analyzed by WB analysis. H3 and GAPDH served as loading controls. (E) Real-time analysis OC expression in response to c-Src inhibition by PP2 in STAT1 depleted cells. MC3T3-E1s were transfected with control siRNA or validated siRNAs targeting STAT1 for 48 hrs and treated with either PP2 (10 μM) or DMSO as controls for 24 hrs. Data shown represent the mean (±SD) of triplicates. One-way ANOVA is conducted; F (3,16) = 7.331, *p* = 0.0026. Bonferroni's multiple comparisons test results *p* = 0.0054, *p* = 0.0428, *p* = 0.0057 for comparing between ctrl (1^st^ bar) and 2^nd^, 3^rd^, and 4^th^ bars, respectively. *p< 0.05, **p<0.01.

Up to date, two distinct mechanisms under which inhibition of SFKs promote osteoblast differentiation have been described and both involve c-Src [35, 37]. The role of Runx2 in osteoblast differentiation has been firmly established [14, 15, 38]. To our best knowledge, to date there are no reports of c-Src directly interacting with Runx2. Another important factor implied in osteogenesis on its own right is STAT1 [16, 17, 39–42]. It has been reported in the past that STAT1 anchors Runx2 in the cytoplasm and decreases its transcriptional activity [43]. Therefore, we investigated the potential inhibitory role of STAT1 downstream of c-Src as an alternative mechanism of describing the inhibitory role of c-Src in osteogenic differentiation. First, STAT1 expression during ES osteogenic differentiation was assessed by WB analysis. Results showed that STAT1 was expressed throughout differentiation (Fig 3A). Next, we examined the subcellular distribution of STAT1 and Runx2 in the absence and presence of c-Src activity by immunofluorescence and found that STAT1 and Runx2 colocalized in synchrony. Runx2 and STAT1 colocalize mainly in the cytoplasm when c-Src is active (when cells are treated with DMSO or PP3). However, after PP2 exposure and inhibition of c-Src activity, the two are separated and while STAT1 remains in the cytoplasm, Runx2 translocates to the nucleus (Fig 3B). Co-expression and colocalization of STAT1 and Runx2 during osteogenic differentiation require their interaction to be tightly regulated. To investigate whether c-Src activity influenced Runx2 interaction with STAT1, day 10 EBs were subjected to IP assay using Runx2 antibody. WB analysis of the immunocomplex showed that upon inhibition of c-Src activity with PP2, Runx2 interaction with STAT1 was significantly reduced (Fig 3C). Similar results were obtained when using specific c-Src siRNA in MC3T3-E1 cells (S6B Fig).

Next, we examined if inhibition of c-Src would still enhance Runx2 nuclear localization when STAT1 was depleted in MC3T3-E1 cells with STAT1 siRNA. The transfection efficiency was tested and optimized in an independent assay (S6C Fig). 48 hours post-transfection, MC3T3-E1s were either treated with DMSO or PP2 for 2 hours prior to fractionation assay. Downregulation of STAT1 with siRNA increased the level of nuclear Runx2 to that in the control, which was MC3T3-E1s treated with PP2. In the absence of STAT1, however, inhibition of c-Src activity with PP2 was not effective in increasing Runx2 nuclear localization (Fig 3D). We further examined Runx2-regulated specific osteogenic marker OC expression in response to STAT1 depletion with and without inhibition of Src activity. OC expression by qPCR analysis confirmed yet again that inhibition of Src activity increased OC expression in the control MC3T3-E1s (second bar in Fig 3E). STAT1 down regulation by itself also increased OC mRNA expression (3^rd^ bar in Fig 3E). However, in the absence of STAT1, Src inhibition by PP2, had no significant effect on OC mRNA abundance (comparing 3^rd^ and 4^th^ bars in Fig 3E). Inhibition of c-Src in STAT1-depleted cells was inefficient in increasing other Runx2-regulated genes including BSPII and COL1A1 (S6D Fig). These results showed that c-Src may exert its inhibitory effect on osteogenesis by an unusual mechanism comprising cytoplasmic anchorage of the major osteogenic factor Runx2 by a minor osteogenic transcription factor STAT1.

STAT1 phosphorylation at Y701 is associated with its increased transcriptional activity [44]. Previously, inhibition of c-Src by PP2 (10 uM) was found to downregulate p-Y701-STAT1 [45]. Therefore, it is possible that phosphorylation of STAT1 downstream of c-Src activity affects its interaction with Runx2. However, an earlier study describing the inhibitory role of STAT1 on Rnux2 transcriptional activity showed that this effect does not depend on STAT1 phosphorylation status at Y701 using mutational assays [43]. Therefore, we speculated other possibilities.

### c-Src enhances STAT1 protein stability

**Fig 4:**
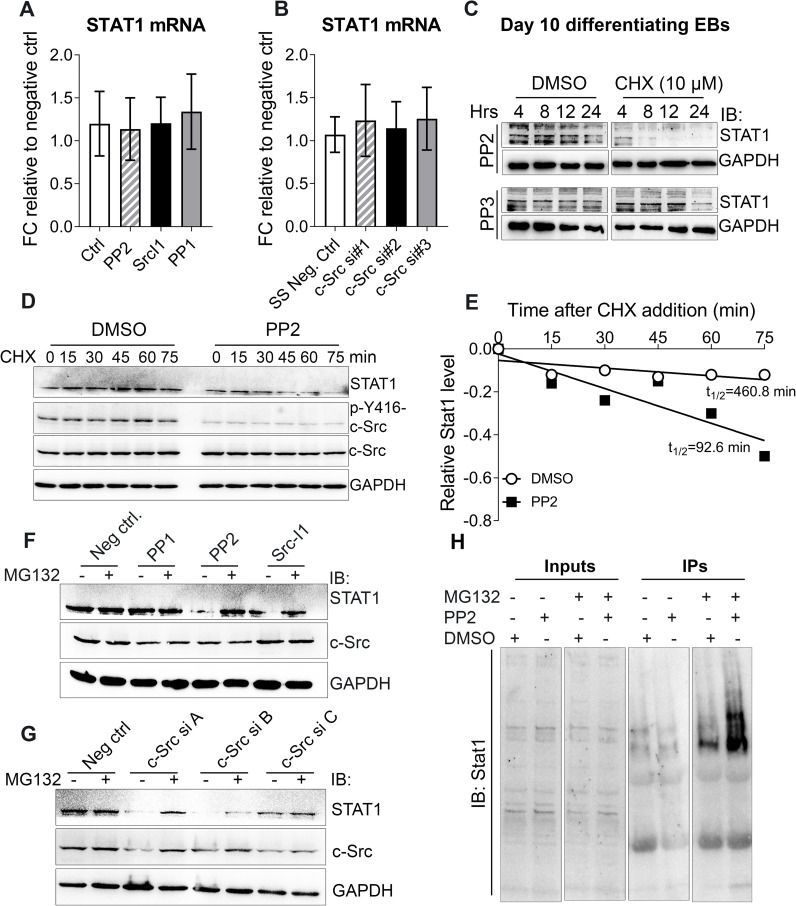
c-Src enhances STAT1 protein stability. (A and B) Real time qPCR analysis of STAT1 expression upon pharmacological inhibition of c-Src using 10 μM of PP1, SrcI-1, and PP2 for 2 hours where DMSO served as negative control (A) and c-Src downregulation using with three different distinct siRNAs where silencer™ select negative siRNA served as control for 48 hours (B) in MC3T3E1 cells. Data shown represent the means (± SD) of triplicates. (C) c-Src inhibition and its effect on STAT1 protein level in mESCs. Differentiating EBs were exposed to 10 μM of PP2 or PP3 for 24 hours prior to treatment with CHX (10 μM) or DMSO. Lysates were collected at the indicated times and subjected to WB analysis using STAT1 antibody. GAPDH served as loading control. (D) Inhibition of c-Src activity and its effect on STAT1 half-life in MC3T3-E1 cells. Cells were subjected to PP2 (10 μM) or DMSO (solvent ctrl) treatments for 2 hrs, prior to addition of CHX (10 uM). Harvested cells at the indicated time points were subjected to WB analysis to examine STAT1 expression. GAPDH served as a loading control. (E) Quantification of STAT1 stability assays. STAT1 band densities were quantified, first normalized to the corresponding GAPDH and then to t = 0 DMSO controls. (F and G) Inhibition of proteasomal degradation by MG132 and its effect on STAT1 loss resulted from c-Src inhibition. MC3T3-E1s were treated with three different c-Src inhibitors including PP2, Src I-1, and PP1 (10 μM) for 24 hrs (F) or were transfected with control siRNA or different combinations of three different specific c-Src siRNA for 48 hrs (A = c-Src siRNAs #1+ #2, B = c-Src siRNAs #1+ #3, and C = c-Src siRNAs #2+ #3) (G). Before harvest, cells were treated with MG132 (10 μM) for 4 hrs as indicated. STAT1, c-Src, and p-c-Src Y416 protein levels were analyzed by immunoblots, with GAPDH as a loading control. (H) Inhibition of c-Src activity and its effect on STAT1 ubiquitination. Proteasomal degradation was inhibited by MG132 in MC3T3-E1 cells for two hours. DMSO served as the negative control. After two hours treated cells underwent second treatments with PP2 (10 μM) or DMSO for another 2 hours. Lysates were subjected to IP assay using STAT1 antibody. Precipitated immunocomplexes were then analyzed by WB using both STAT1 antibody.

Interaction of STAT1 and Runx2 is independent of STAT1 phosphorylation status at Y701 [43]. Therefore, other regulatory mechanisms may exist, which do not involve tyrosine phosphorylation modifications of STAT1. We first sought to assess whether c-Src activity would impact STAT1 expression. qPCR analysis of lysates treated with three different Src inhibitors, PP2, PP1, and SrcI1 showed no statistical differences in the abundance of STAT1 message (Fig 4A). The same results were obtained in Src-depleted MC3T3-E1s using specific c-Src siRNAs (Fig 4B).

The homeostasis of protein metabolism is maintained and regulated by the rates of its biosynthesis and degradation. To determine whether c-Src affects STAT1 turnover, we investigated the half-life of STAT1 in the presence or absence of c-Src activity when STAT1 biosynthesis was inhibited by cycloheximide (CHX; 10 μM). WB analysis of PP2-treated mESCs revealed decreased level of STAT1 protein in response to protein biosynthesis inhibition at the earliest collection time. However, in mESCs treated for 24 h with PP3 (inactive control) in the presence of CHX, STAT1 was still detectable at the level comparable to CHX-free condition (Fig 4C). Tighter time-course analysis revealed that pharmacological inhibition of c-Src significantly shortened the half-life of endogenous STAT1 in MC3T3-E1 (Fig 4D and 4E) supporting that c-Src regulates STAT1 mainly through enhancing protein stability. The effect of c-Src activity on STAT1 half-life raised the question whether or not c-Src regulates protein stability of STAT1 through proteasomal degradation. To address this question, we examined STAT1 protein level in the absence and presence of MG132, a 26S proteasome inhibitor, when c-Src activity was inhibited in MC3T3-E1 cells. MG132 rescued STAT1 loss caused by c-Src inhibition (Fig 4F), arguing that c-Src regulates STAT1 expression primarily via a posttranslational mechanism. Results were confirmed using c-Src specific siRNAs (Fig 4G). Ubiquitin-proteasome pathway is one the major mechanism for the regulation of activated STAT1 [43]. Therefore, ubiquitination of STAT1 was assessed in an immunoprecipitation assay where c-Src was inhibited by PP2 (10 μM) in MC3T3-E1 cells. WB analysis of precipitated immunocomplexes revealed accumulation of ubiquitinated STAT1 in cell lysates, in which c-Src activity and proteasomal degradation were inhibited (Fig 4H).Collectively, our results strongly suggest a regulatory role of c-Src in regulation of Stat1 stability through proteasome-ubiquitination pathway. However, the mechanisms under which c-Src down regulates STAT1 ubiquitination remains to be investigated. Human homologof Drosophila Seven-In-Absentia (SIAH2) has been previously identified as a ubiquitin ligase that stabilizes STAT1 by downregulation of TYK2, a kinase responsible for STAT1 phosphorylation and its subsequent nuclear localization [46]. Phosphorylation and activation of SIAH2 by c-Src has been demonstrated by others [47]. Therefore, one possible mechanism to investigate could be the increase of cytoplasmic STAT1 stability through regulation of SIAH2 activity by c-Src. In summary, we show that c-Src regulates the interaction of STAT1 with Runx2, which occurs in the cytoplasm. Moreover, we demonstrate that c-Src activity decreases STAT1 proteasomal degradation through ubiquitination. Inhibition of c-Src activity ablates the interaction of Runx2 with STAT1 and permits the shuttling of Runx2 to the nucleus. These findings provide insights into a novel mechanism of STAT1-Runx2 interaction and the inhibitory role of a proto-oncogene, c-Src, in osteogenic differentiation of mESCs *in vitro*.

## Supporting information

S1 Fig mRNA expression of osteogenic-related markers during 21 days of osteogenic differentiation from mESCs. Gene expression of Runx2, Osx, BSPII, COL1A1, and OC of ESCs (day 0) and differentiating cells (day1 to 21) which were induced for osteogenic differentiation were analyzed by real time qPCR using the primer pairs listed in S1 Table. Data shown represent the mean (±SD) of triplicates.
(PDF)

S2 Fig PP2 (10 μM) effectively downregulates p-Y416-c-Src. (A and B) Three different inhibitors of c-Src were applied in the concentrations of 1μM and 10 μM in day 5 differentiating EBs for 2 and 24 hours. Lysates were collected and subjected to immunoblot analysis using p-Y416-c-Src and c-Src antibodies. GAPDH served as loading control. (C) Day 5 differentiating EBs were treated with different concentrations of PP2, and PP3 (inactive analog PP2) of including 0.1, 1, 5, 10, and 20 μM for 2 hours. Adjusted volume of DMSO for each corresponding concentration of PP2 served as the solvent control. Lysates were prepared and subjected to immunoblot analysis using p-Y416-c-Src and c-Src antibodies. GAPDH served as loading control. (D) Scheme shows eight different periods in which activity of c-Src was inhibited by PP2 (10 μM) during 21-day osteogenic differentiation protocol from mESCs. (E) Alizarin Red S Staining (ARS) of day 21 osteo-nodules treated with PP2 (10 μM) for the indicated time periods. Mineralization of osteo-nodules at the end of differentiation is assessed by ARS staining.
(PDF)

S3 Fig Transfection of mES R1 cells was inefficient. Different c-Src siRNA from Ambion (A) and Thermo Fisher Scientific (B) along with GAPDH specific siRNA as a positive control were applied using transfectamin to downregulate c-Src activity in mESCs for the indicated concentrations. Lysates were subjected to WB analysis using c-Src antibody. GAPDH served as loading control. (C) mESCs were subjected to transfection using electroporation with the indicated concentrations of c-Src and GAPDH specific siRNAs. Lysates were analyzed by WB using c-Src and GAPDH antibodies.
(PDF)

S4 Fig c-Src downregulation using specific siRNA or its inhibition by PP2 increased osteogenic differentiation in MC3T3-E1s. (A) Transfection efficiency of the applied specific c-Src siRNA were examined by WB. (B) OC mRNA expression at day 14 and 21 of differentiation when c-Src is depleted in MC3T3-E1s using specific c-Src siRNA. Data shown represent the mean (±SD) of triplicates. Unpaired two-tailed t test is performed (*p* = 0.049). (C) ARS analysis of Src depleted MC3T3-E1s by c-Src specific siRNAs. Day 14 and 21 Src depleted differentiating MC3T3-E1 cells were subjected to ARS analysis. Quantified values were normalized against their corresponding DMSO and graphed. Data represents the means (±SD) of triplicates. Unpaired two-tailed t test is performed (*p* = 0.0074 for day 14 comparison and *p* = 0.0037 for day 21). (D) Day 21 OC mRNA expression in response to c-Src inhibition in MC3T3-E1 by different dosages of PP2. OC mRNA expression in response to different dosages of PP2 was measured by real time qPCR and normalized to the corresponding DMSO controls. Data represents the mean (±SD) of triplicates. One-way ANOVA was conducted; F = 19.49, *p*<0.0001. Tukey's multiple comparisons test indicated significant differences between DMSO and PP2 (10 uM) with *p*<0.0001, PP2 (1 uM) and 10 uM with *p*<0.0001, and PP2 (5 uM) and 10 uM with *p* = 0.0009. (E) Day 21 osteo nodules mineralization in response to c-Src inhibition in MC3T3-E1 by different dosages of PP2. Absorbed Alizarin red stain in response to different dosages of PP2 was measured and normalized to DMSO controls. Data represents the mean (±SD) of triplicates. One-way ANOVA was conducted; F = 8.589, *p* = 0.007. Tukey's multiple comparisons test indicated significant differences between DMSO and PP2 (10 uM) with *p* = 0.0098, PP2 (1 uM) and 10 uM with *p* = 0.012, and PP2 (5 uM) and 10 uM with *p* = 0.028. (F) COL2A mRNA expression in response to c-Src inhibition by PP2. MC3T3-E1 cells were treated with 1, 5, 10, and 20 μM PP2. Lysates were analyzed by real time qPCR. Data shown represent the means (±SD) of triplicates. *p< 0.05, **p<0.01, ***p<0.005, and ****p<0.0001.
(PDF)

S5 Fig Examining efficacy of constitutively active c-Src over-expression in MC3T3-E1s. (A) Lenti-X 293T cell line was co-transfected with dox-inducible EGFP linked lentiviral vector expressing constitutively active c-Src along with ectopic Lenti-X Packaging Single Shot packaging plasmid for 4 hours. Cells were imaged 48 hours post-transfection with and without doxycycline. (B) Transduced cells for the indicated conditions were lysed 48 hours after transfection and were subjected to WB analysis using Y416 phospho-specific c-Src, and c-Src antibodies. GAPDH served as loading control.
(PDF)

S6 Fig c-Src regulates Runx2 subcellular localization through STAT1. (A) Runx2 nuclear fractions in MC3T3-E1 c-Src depleted cells. Nuclear extracts were subjected to immunoblot analysis with GAPDH (cytosolic marker) and H3 (nuclear marker) as loading controls. (B) Runx2 interaction with STAT1 in c-Src depleted cells. 48 hrs post-transfection, MC3T3-E1s transfected with c-Src specific siRNA were lysed and subjected to IP using STAT1 antibody. Precipitated immunocomplexes were then subjected to WB analysis using Runx2 and STAT1 antibodies. (C) Examining STAT1 expression level post-transfection with specific STAT1 siRNA. GAPDH and SS negative ctrl siRNAs served as positive and negative controls, respectively. (D) Osteogenic marker expression in the absence and presence of c-Src activity in STAT1 depleted cells. STAT1 depleted MC3T3-E1s were treated with either PP2 (10 μM) or DMSO as control for 24 hrs. Cell lysates were subjected to qPCR analysis and graphed.
(PDF)

S1 Table Primer pairs used in qPCR analysis. (TIF)

S2 Table Primer pairs used in ChIP analysis. (TIF)

S1 Raw images (PDF)
