# Supplementary figures and images for: c-Src kinase inhibits osteogenic differentiation via enhancing STAT1 stability
Source: PLoS One. 2020 Nov 12;15(11):e0241646. doi: 10.1371/journal.pone.0241646 (PMC7660501; doi:10.1371/journal.pone.0241646)

**Figure S1**

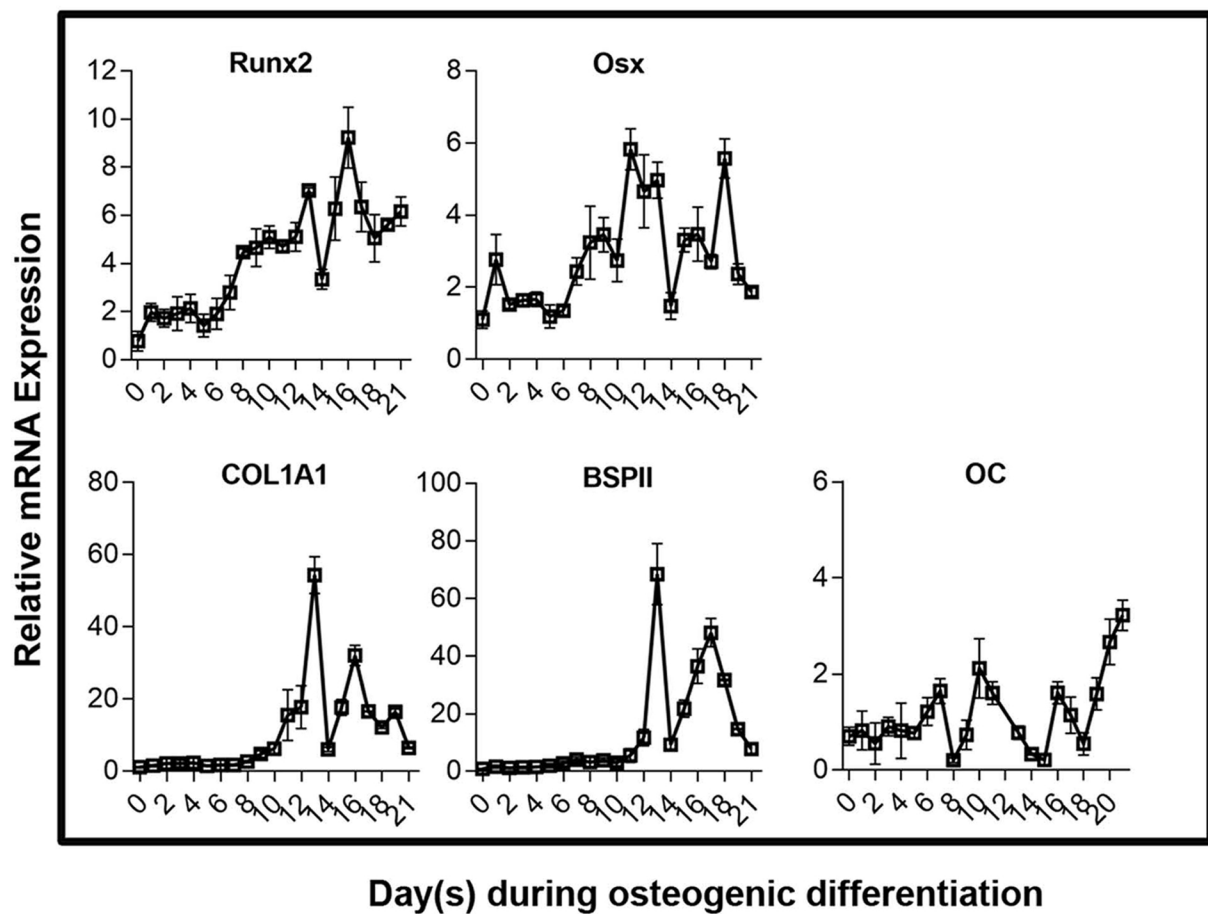

Supplement: S1 Fig — Gene expression of Runx2, Osx, BSPII, COL1A1, and OC of ESCs (day 0) and differentiating cells (day1 to 21) which were induced for osteogenic differentiation were analyzed by real time qPCR using the primer pairs listed in S1 Table. Data shown represent the mean (±SD) of triplicates. (PDF) [file pone.0241646.s001.pdf]

Figure S2

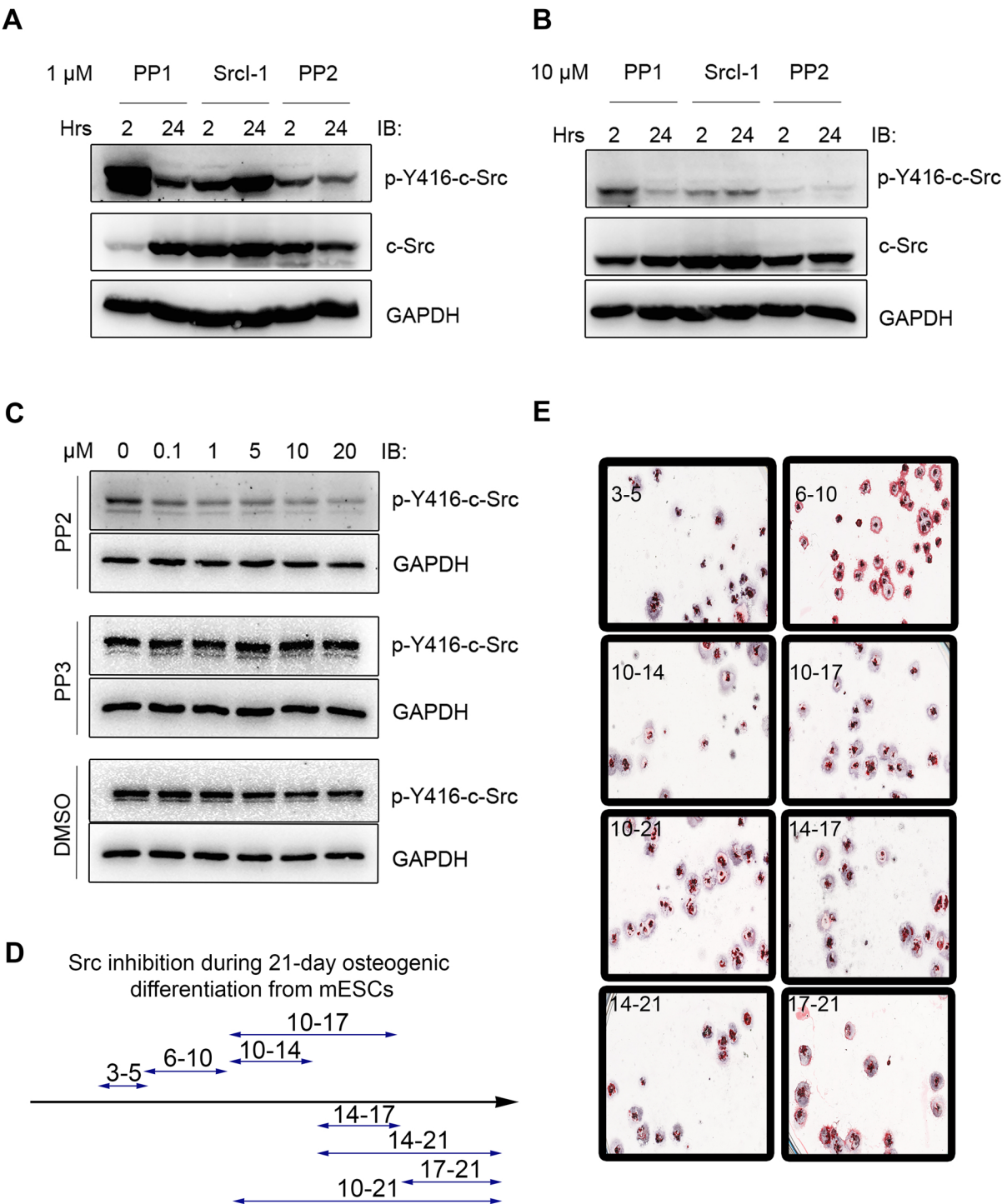

Supplement: S2 Fig — (A and B) Three different inhibitors of c-Src were applied in the concentrations of 1μM and 10 μM in day 5 differentiating EBs for 2 and 24 hours. Lysates were collected and subjected to immunoblot analysis using p-Y416-c-Src and c-Src antibodies. GAPDH served as loading control. (C) Day 5 differentiating EBs were treated with different concentrations of PP2, and PP3 (inactive analog PP2) of including 0.1, 1, 5, 10, and 20 μM for 2 hours. Adjusted volume of DMSO for each corresponding concentration of PP2 served as the solvent control. Lysates were prepared and subjected to immunoblot analysis using p-Y416-c-Src and c-Src antibodies. GAPDH served as loading control. (D) Scheme shows eight different periods in which activity of c-Src was inhibited by PP2 (10 μM) during 21-day osteogenic differentiation protocol from mESCs. (E) Alizarin Red S Staining (ARS) of day 21 osteo-nodules treated with PP2 (10 μM) for the indicated time periods. Mineralization of osteo-nodules at the end of differentiation is assessed by ARS staining. (PDF) [file pone.0241646.s002.pdf]

**Figure S3**

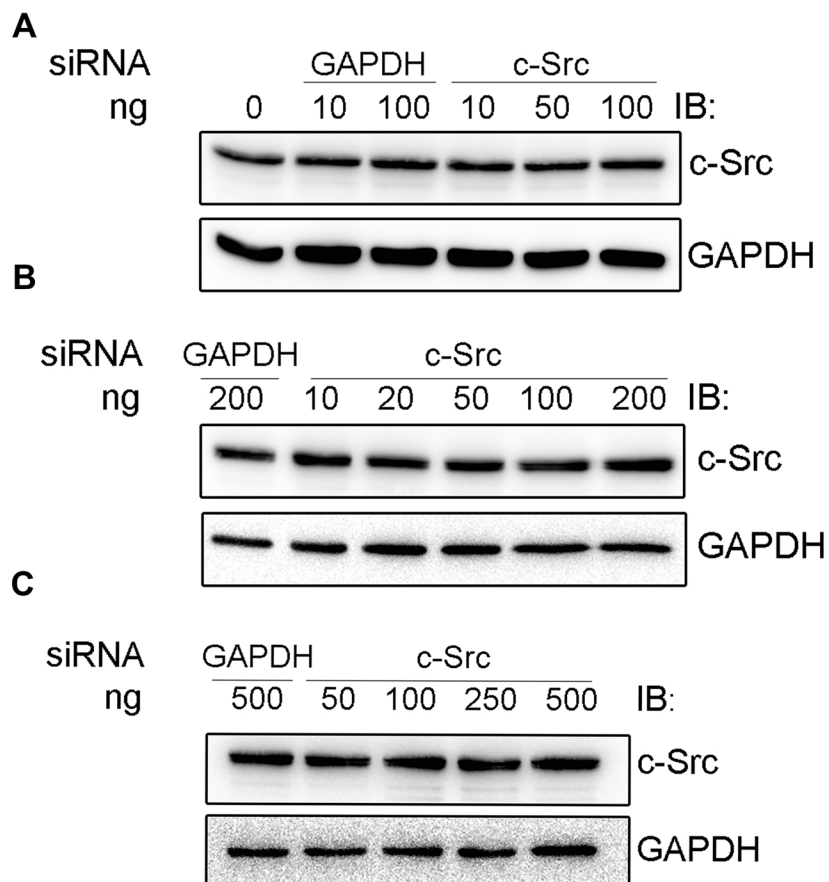

Supplement: S3 Fig — Different c-Src siRNA from Ambion (A) and Thermo Fisher Scientific (B) along with GAPDH specific siRNA as a positive control were applied using transfectamin to downregulate c-Src activity in mESCs for the indicated concentrations. Lysates were subjected to WB analysis using c-Src antibody. GAPDH served as loading control. (C) mESCs were subjected to transfection using electroporation with the indicated concentrations of c-Src and GAPDH specific siRNAs. Lysates were analyzed by WB using c-Src and GAPDH antibodies. (PDF) [file pone.0241646.s003.pdf]

Figure S4

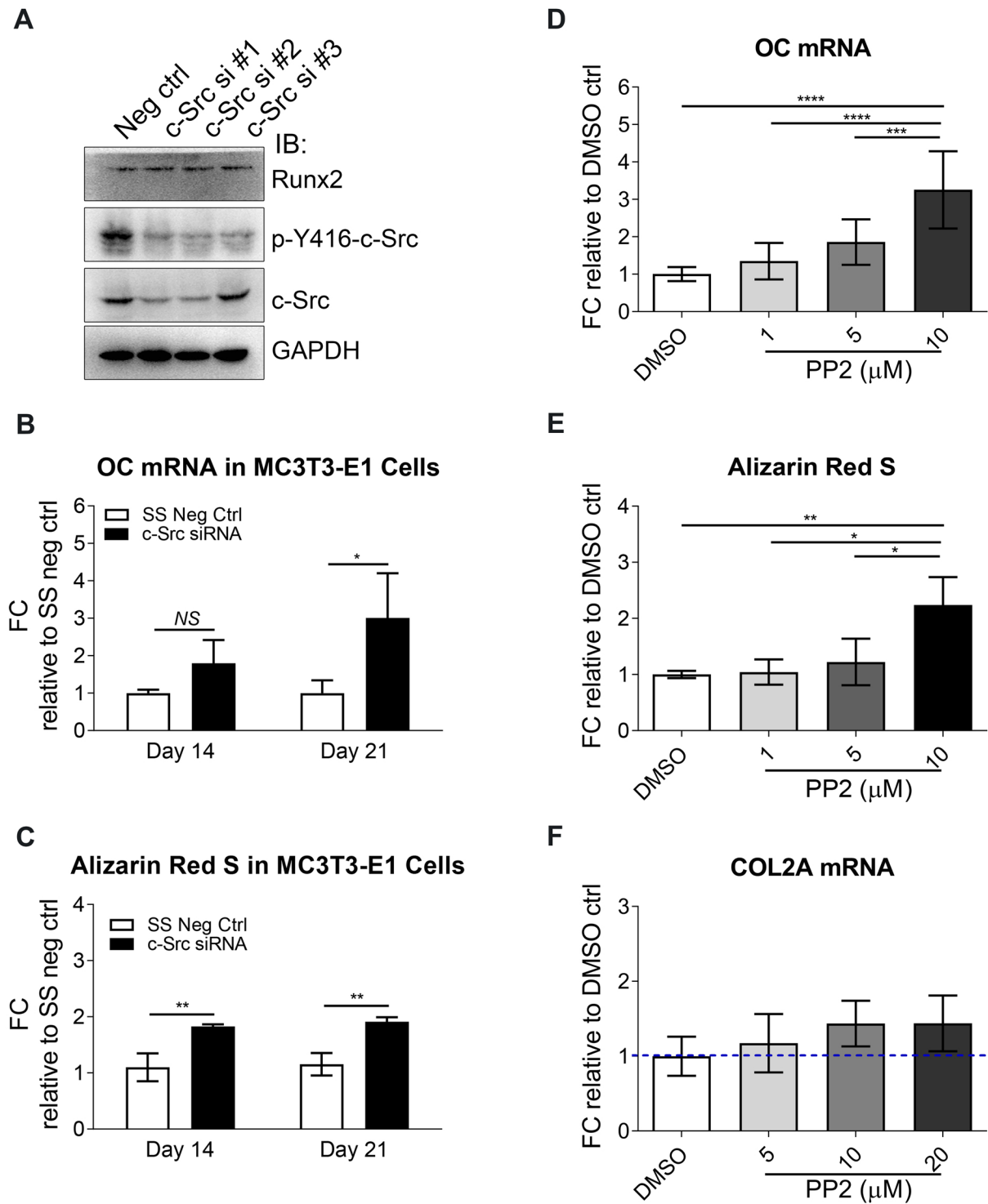

Supplement: S4 Fig — (A) Transfection efficiency of the applied specific c-Src siRNA were examined by WB. (B) OC mRNA expression at day 14 and 21 of differentiation when c-Src is depleted in MC3T3-E1s using specific c-Src siRNA. Data shown represent the mean (±SD) of triplicates. Unpaired two-tailed t test is performed (p = 0.049). (C) ARS analysis of Src depleted MC3T3-E1s by c-Src specific siRNAs. Day 14 and 21 Src depleted differentiating MC3T3-E1 cells were subjected to ARS analysis. Quantified values were normalized against their corresponding DMSO and graphed. Data represents the means (±SD) of triplicates. Unpaired two-tailed t test is performed (p = 0.0074 for day 14 comparison and p = 0.0037 for day 21). (D) Day 21 OC mRNA expression in response to c-Src inhibition in MC3T3-E1 by different dosages of PP2. OC mRNA expression in response to different dosages of PP2 was measured by real time qPCR and normalized to the corresponding DMSO controls. Data represents the mean (±SD) of triplicates. One-way ANOVA was conducted; F = 19.49, p<0.0001. Tukey's multiple comparisons test indicated significant differences between DMSO and PP2 (10 uM) with p<0.0001, PP2 (1 uM) and 10 uM with p<0.0001, and PP2 (5 uM) and 10 uM with p = 0.0009. (E) Day 21 osteo nodules mineralization in response to c-Src inhibition in MC3T3-E1 by different dosages of PP2. Absorbed Alizarin red stain in response to different dosages of PP2 was measured and normalized to DMSO controls. Data represents the mean (±SD) of triplicates. One-way ANOVA was conducted; F = 8.589, p = 0.007. Tukey's multiple comparisons test indicated significant differences between DMSO and PP2 (10 uM) with p = 0.0098, PP2 (1 uM) and 10 uM with p = 0.012, and PP2 (5 uM) and 10 uM with p = 0.028. (F) COL2A mRNA expression in response to c-Src inhibition by PP2. MC3T3-E1 cells were treated with 1, 5, 10, and 20 μM PP2. Lysates were analyzed by real time qPCR. Data shown represent the means (±SD) of triplicates. *p< 0.05, **p<0.01, ***p<0.005, [file pone.0241646.s004.pdf]

Figure S5

A

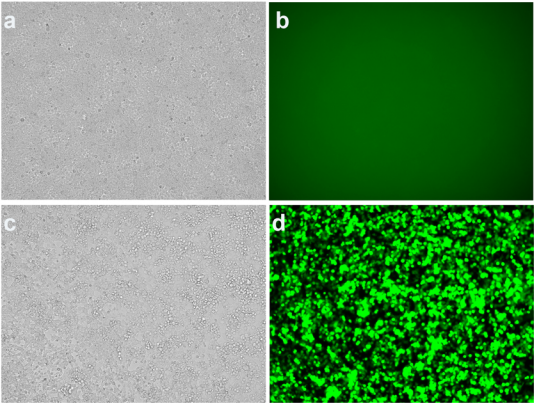

B

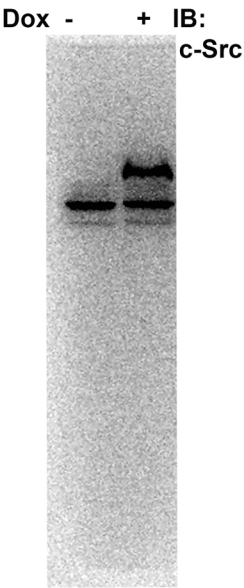

Supplement: S5 Fig — (A) Lenti-X 293T cell line was co-transfected with dox-inducible EGFP linked lentiviral vector expressing constitutively active c-Src along with ectopic Lenti-X Packaging Single Shot packaging plasmid for 4 hours. Cells were imaged 48 hours post-transfection with and without doxycycline. (B) Transduced cells for the indicated conditions were lysed 48 hours after transfection and were subjected to WB analysis using Y416 phospho-specific c-Src, and c-Src antibodies. GAPDH served as loading control. (PDF) [file pone.0241646.s005.pdf]

**Figure S6**

**A**

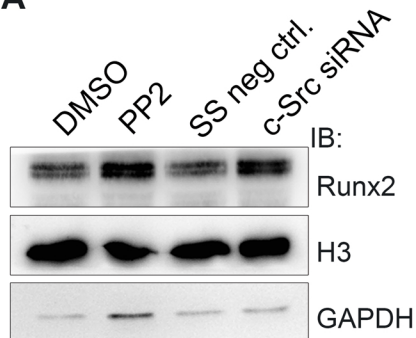

**C**

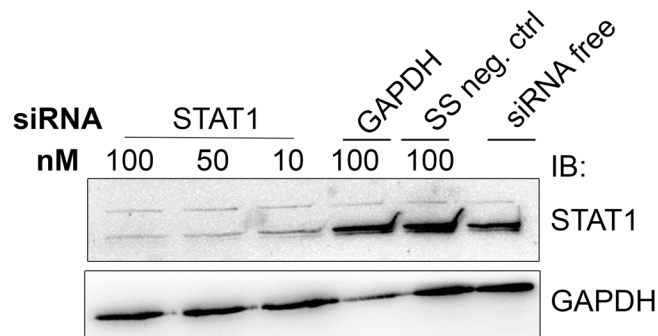

**B**

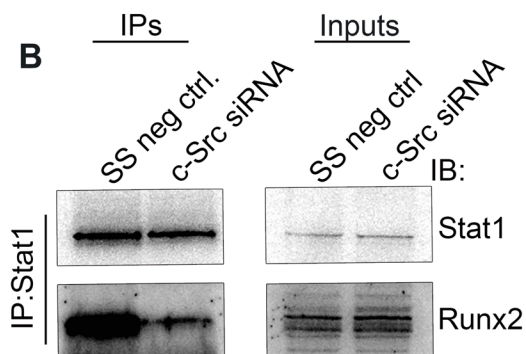

**D**

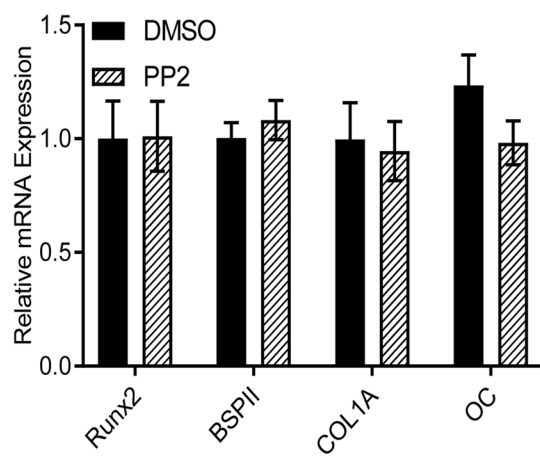

Supplement: S6 Fig — (A) Runx2 nuclear fractions in MC3T3-E1 c-Src depleted cells. Nuclear extracts were subjected to immunoblot analysis with GAPDH (cytosolic marker) and H3 (nuclear marker) as loading controls. (B) Runx2 interaction with STAT1 in c-Src depleted cells. 48 hrs post-transfection, MC3T3-E1s transfected with c-Src specific siRNA were lysed and subjected to IP using STAT1 antibody. Precipitated immunocomplexes were then subjected to WB analysis using Runx2 and STAT1 antibodies. (C) Examining STAT1 expression level post-transfection with specific STAT1 siRNA. GAPDH and SS negative ctrl siRNAs served as positive and negative controls, respectively. (D) Osteogenic marker expression in the absence and presence of c-Src activity in STAT1 depleted cells. STAT1 depleted MC3T3-E1s were treated with either PP2 (10 μM) or DMSO as control for 24 hrs. Cell lysates were subjected to qPCR analysis and graphed. (PDF) [file pone.0241646.s006.pdf]

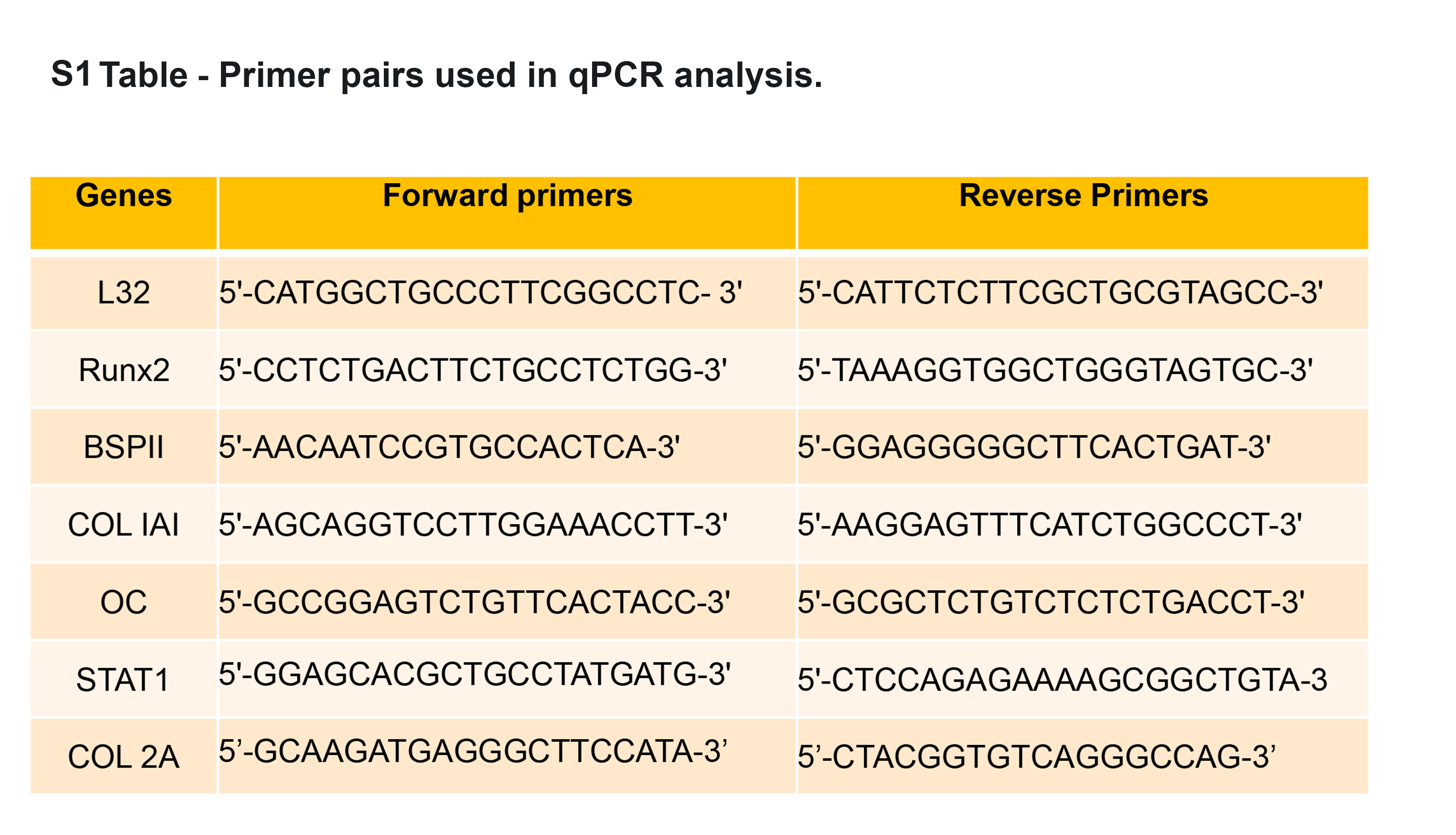

Supplement: S1 Table — (TIF) [file pone.0241646.s007.tif]

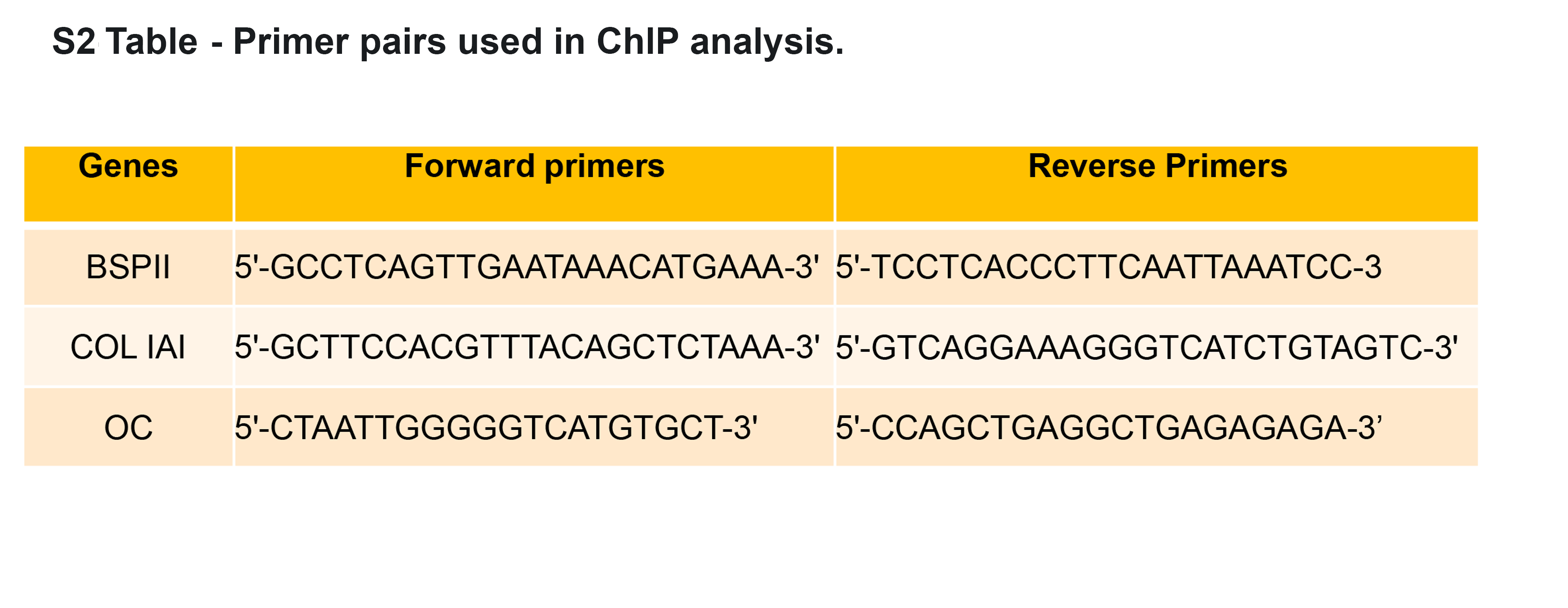

Supplement: S2 Table — (TIF) [file pone.0241646.s008.tif]
