# Supplementary material for: c-Src kinase inhibits osteogenic differentiation via enhancing STAT1 stability
Source: PLoS One. 2020 Nov 12;15(11):e0241646. doi: 10.1371/journal.pone.0241646 (PMC7660501; doi:10.1371/journal.pone.0241646)

Fig. 1A.

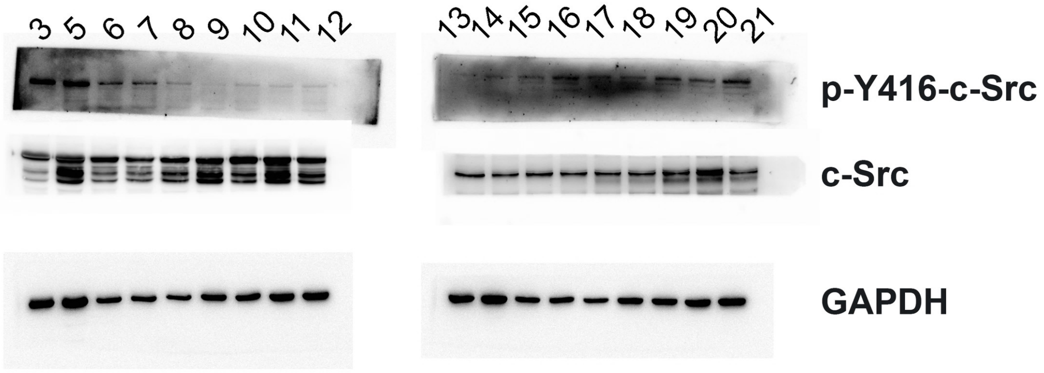

Fig. 2D

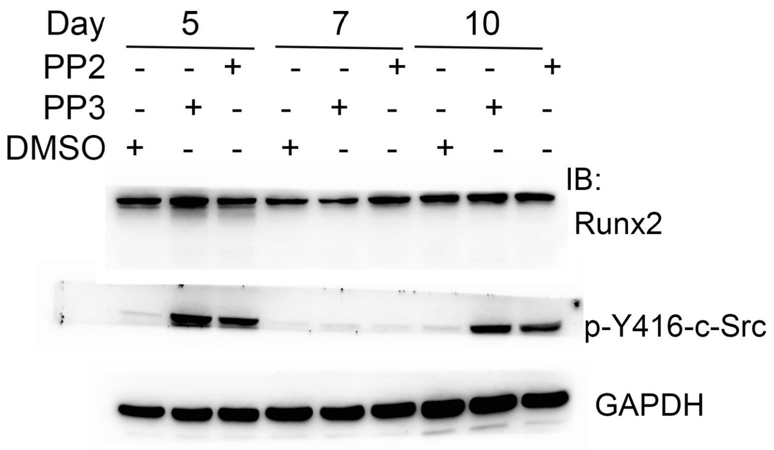

All blots for figure 2D were reverted on the original figure to show the orders from left to right.

Fig. 2E

In Figure 2E, originally we looked into multiple time points for such assay. The last three samples are shown in the original MS refelecting the indicated day of sampling described in the MS.

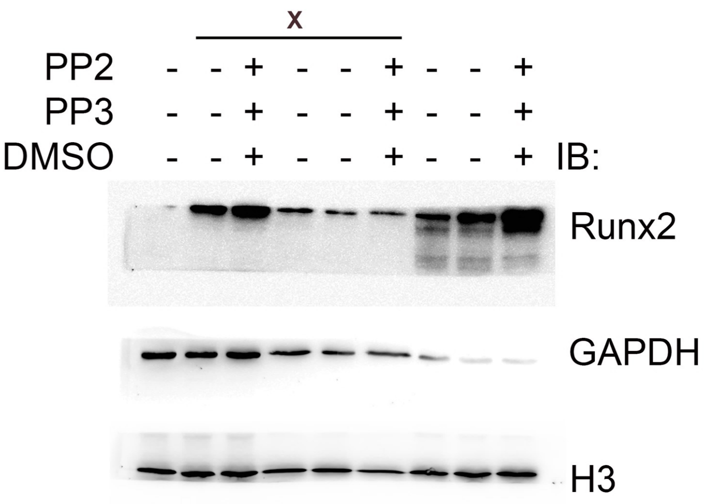

Fig. 3A

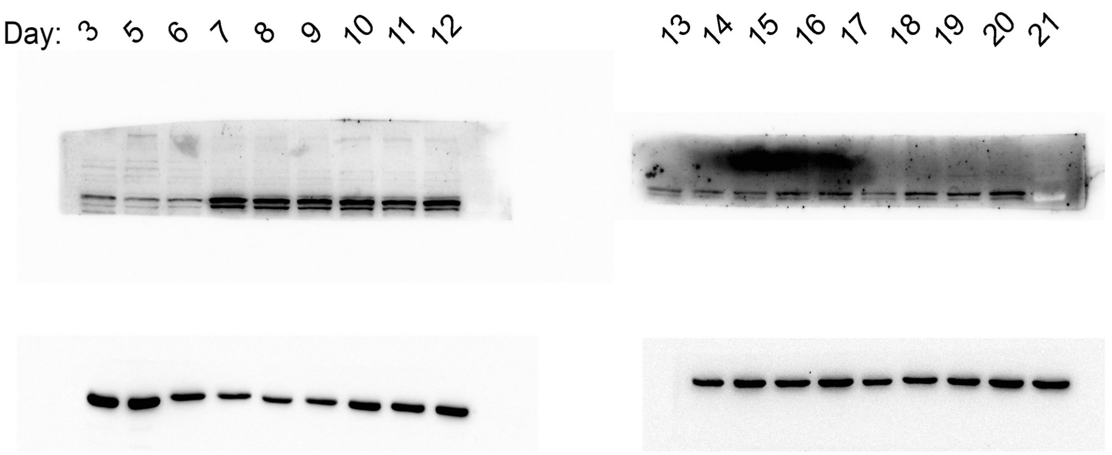

Fig. 3C

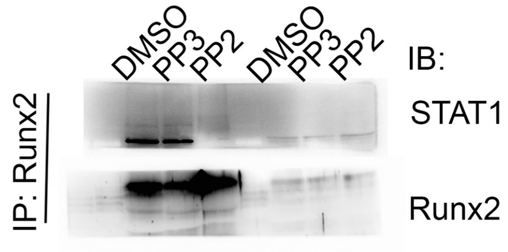

Fig. 3D

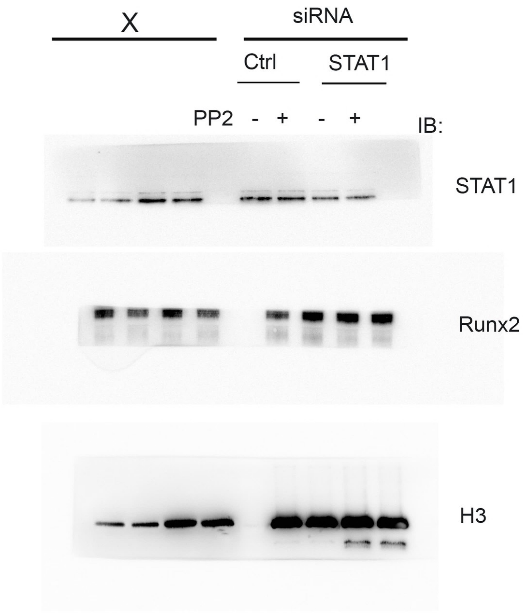

Fig. 4C

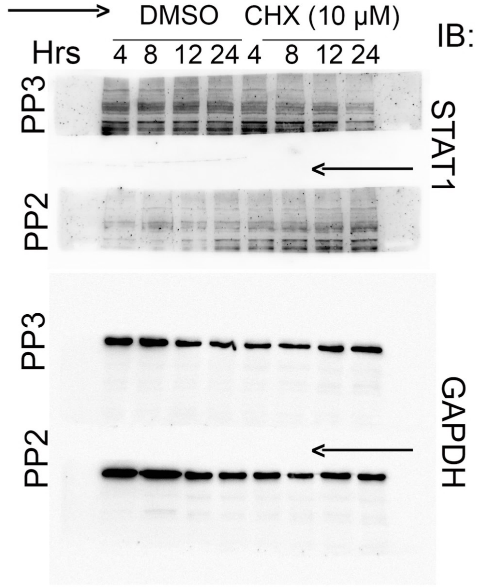

PP2 panels for Stat1 and GAPDH are flipped in fig 4C to keep the orders consistent.

Fig. 4F

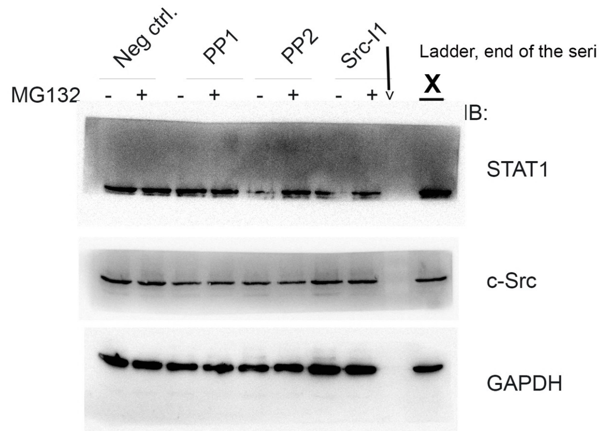

Fig. 4G

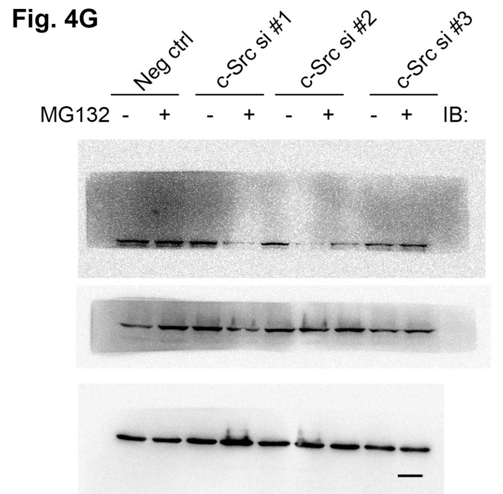

X: both x in 4F and 4G refers to a ctrl that is not included in the figure.

Fig. 4D

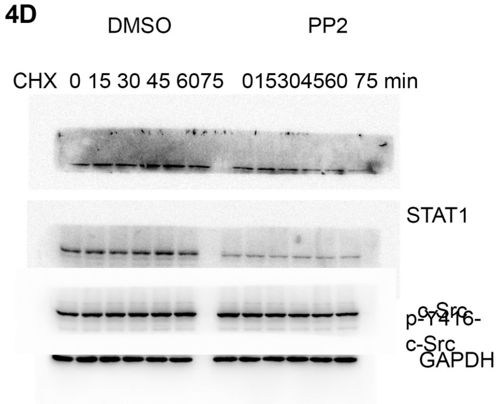

Fig. 4H

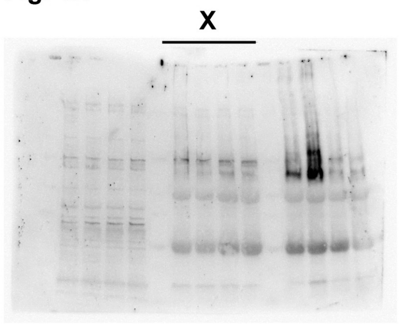

Figure S2-A & B: A replicate from set of samples on the left side were not shown in the MS.

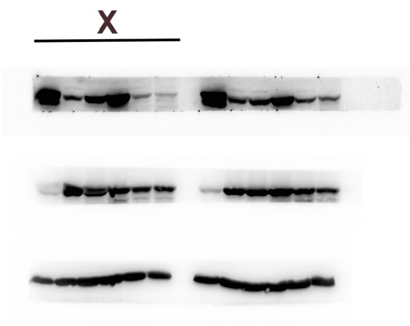

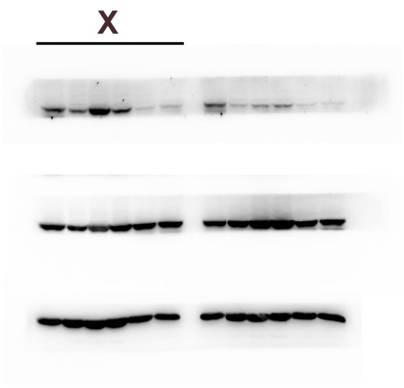

Fig. S2C

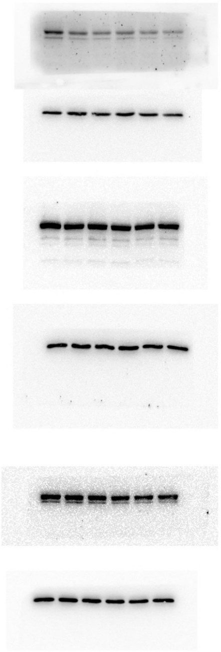

Fig. S3A

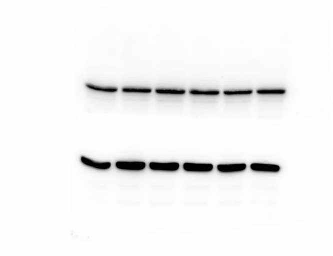

c-Src

GAPDH

Fig. S3B

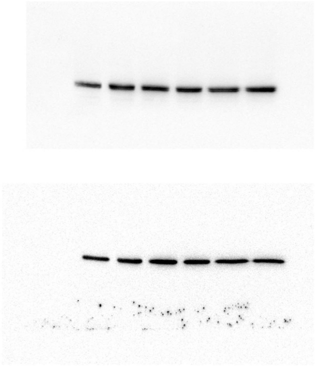

c-Src

GAPDH

Fig. S3B

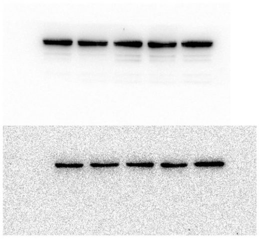

c-Src

GAPDH

All blots are as the same order as presented in the original figure.

Fig. S3D

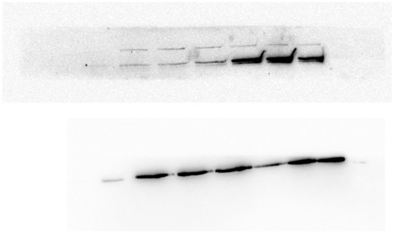

STAT1

GAPDH

Figure S4- the last blot represent GAPDH as loading ctrl. Since Runx2 and c-Src, and p-c-Src-Y416 are all about 60 kd, the samples were loaded 3x to have enough membranes to avoid stripping and re-blotting. In this MS we used the first set of GAPDH.

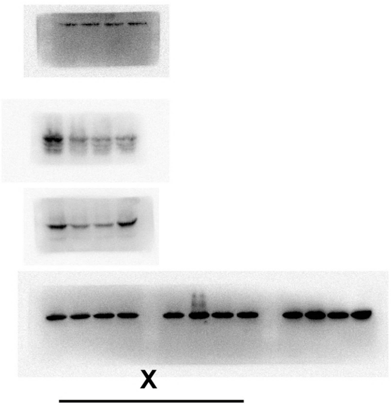

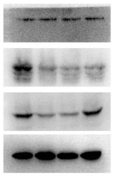

Fig. S5A

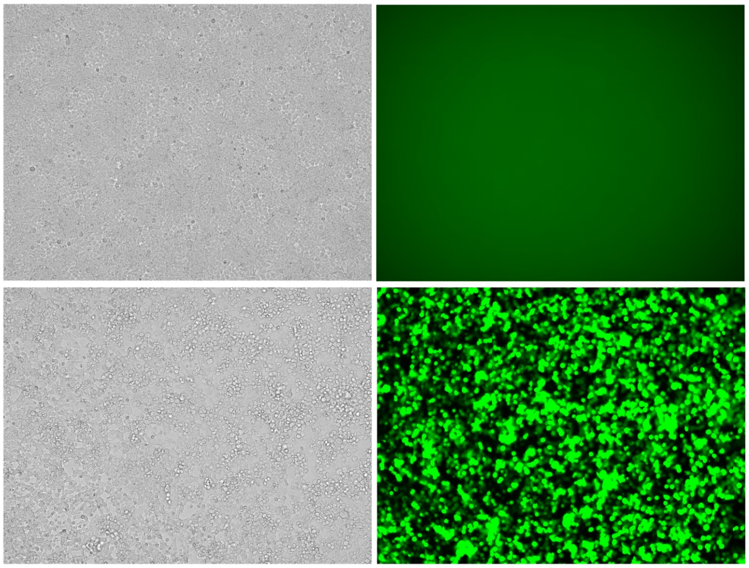

Fig. S5B: the blot was reverted to show the negative ctrl first.

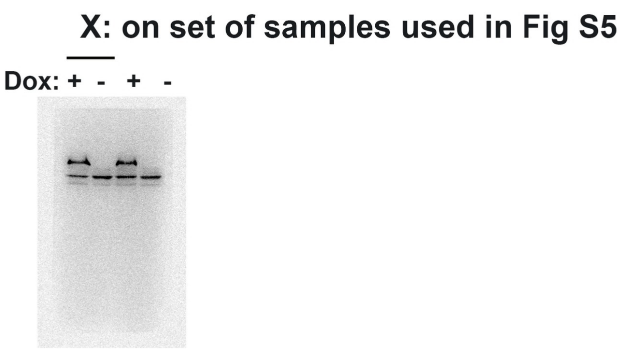

Fig. S6A: The left set of samples represents what we showed in MS. All three membranes were reverted in the MS.

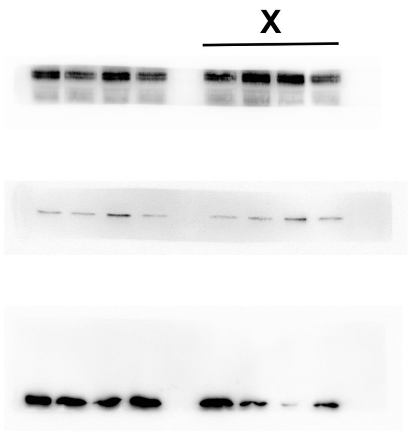

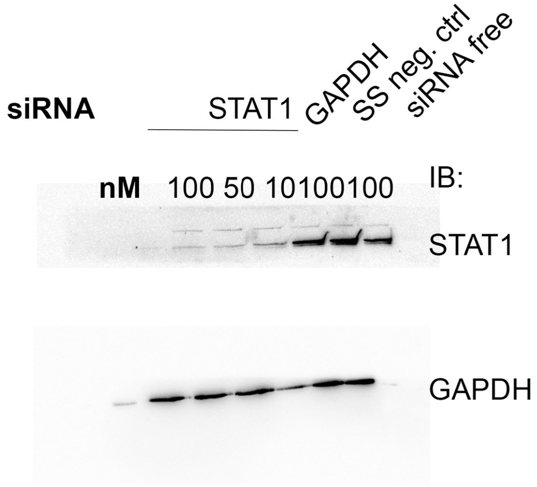

Fig. S6B: Different exposure times for each membrane are available in case needed!

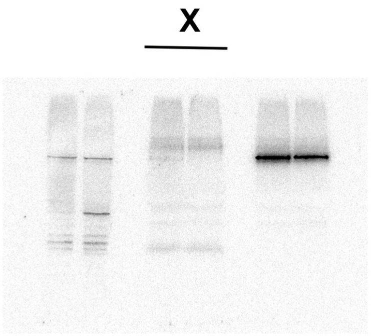

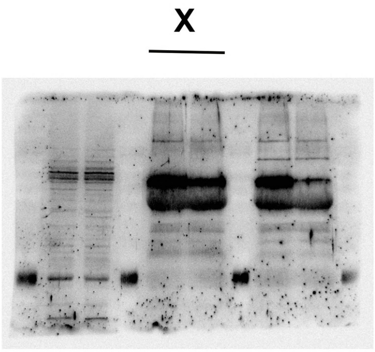

Supplement: S1 Raw images — (PDF) [file pone.0241646.s009.pdf]
